# Thirty-two new and noteworthy floristic records from north-eastern Greece

**DOI:** 10.3897/BDJ.10.e81817

**Published:** 2022-04-21

**Authors:** Panayiotis Doumas, Katerina Goula, Theophanis Constantinidis

**Affiliations:** 1 8th Elementary School, Xanthi, Greece 8th Elementary School Xanthi Greece; 2 Section of Ecology & Systematics, Department of Biology, National and Kapodistrian University of Athens, Athens, Greece Section of Ecology & Systematics, Department of Biology, National and Kapodistrian University of Athens Athens Greece

**Keywords:** Balkan countries, endemics, flora, new records, Macedonia, Thrace

## Abstract

**Background:**

The vascular flora of Greece comprises no less than 6,705 vascular plant taxa (species and subspecies, including established aliens) in an area of 131,957 km^2^. The phytogeographic region of north-east Greece (NE), an area characterised by physiographic diversity, is the richest floristic region in Greece, yet it remains less-explored compared to other regions.

**New information:**

We present an annotated inventory of 32 vascular plant taxa collected predominantly from the East Macedonia and Thrace Regions (Makedonia and Thraki, NE Greece), following fieldwork that took place between 2013 and 2021. In our results, we identify seven new additions to the Greek flora (Aethionemasaxatilesubsp.rhodopaeum, *Anthericumramosum*, *Asteramellus*, *Euphorbiacarniolica*, Hesperismatronalissubsp.matronalis, *Saponariastranjensis* and *Silenefetlerii*) and 25 new records to the regional Greek flora consisting of mostly rare Balkan endemics of a restricted distribution and presumable conservation priorities. Taxonomic comments are added, where necessary. IUCN threat categories are assigned to certain taxa. Our investigation highlights the importance of the Balkan border areas as a source of new floristic elements for single countries and points to the importance of range-restricted taxa, in addition to single-country endemics, to address plants with a narrow distribution pattern.

## Introduction

Greece is a Mediterranean country with a rich flora. According to current estimates, no less than 6,705 vascular plant taxa (species and subspecies, including established aliens) grow in an area of 131,957 km^2^ (Dimopoulos 2018). This taxa number results from a rather conservative taxonomic approach, particularly in the case of some critical genera (e.g. *Allium*, *Limonium*, *Οphrys*), where species delimitation is problematic (Strid and Tan 2017). The degree of floristic exploration of various Greek regions is rather uneven. The Island of Kriti (Crete, Cr) to the south is amongst the most well-explored areas, whereas the far northeast (Eastern Makedonia and Thraki, NE) belongs to the less-explored areas, despite the large number of taxa already documented in this region (Strid and Tan 2017; Dimopoulos 2018).

The territory of Thraki (Thrace) in particular, often reported as a single floristic entity in the early floristic and taxonomic literature (e.g. Hayek 1927), is nowadays divided amongst three different countries: Bulgaria, Greece and the European part of Turkey. The Pirin, Rila and Rodopi mountain ranges dominate in its western parts with a mosaic of different habitats. Hills and the Thrakian plains mostly characterise the eastern parts of the area. Greece owns the south-western parts of Thraki and some noteworthy limestone mountains (Falakro, Menikio, Orvilos) are predominant to the west of that area. The floristic connections of the wider Thraki area are reflected by the distribution of several taxa restricted in this region: *Centaureaparilica* Stoj. & Stef., *Chondrillaurumoffii* Degen, *Fritillariadrenovskii* Degen & Stoj., *Haberlearhodopensis* Friv., *Liliumrhodopeum* Delip., *Salixxanthicola* K.I. Chr., *Soldanellarhodopaea* F.K. Mey. and *Violaperinensis* W. Becker are examples of taxa shared between Bulgaria and Greece, while *Hypericumthasium* Griseb., *Verbascumadrianopolitanum* Podp. and *V.humile* Janka are further distributed to parts of European Turkey (Petrova and Vladimirov 2010). *Carduusthracicus* (Velen.) Hayek, *Heptapteratriquetra* (Vent.) Tutin, *Hypecoumponticum* Velen., *Onosmathracica* Velen. and *Veronicaturrilliana* Stoj. & Stef. are examples of endemic species shared between Bulgaria and European Turkey (Petrova and Vladimirov 2010), while *Dianthusingoldbyi* Turrill and *Bellevaliaedirnensis* Özhatay & B. Mathew (Strid and Tan 1997, Bareka 2007) are rare, local species shared between Greece and European Turkey.

As a rule, the border areas of Greece with the countries to the north (Albania, North Macedonia and Bulgaria) are formed by high mountains encompassing natural habitats covered by extensive forests. The north-eastern border area with European Turkey is mostly hilly and of low altitude, sustaining cultivation and stock-farming. The turbulent past of this region and its rugged terrain are some of the parameters responsible for retaining the area less well-explored floristically, compared to other Greek regions. As a result, new biodiversity records from the north continue to enrich the flora of Greece (e.g. Tsiftis et al. 2019) and new discoveries are to be expected in the future.

## Materials and methods

Our study is based on field collections made between 2013 and 2021. For the identification of the plant specimens and their distinction from related taxa, we used the relevant treatments in basic floristic works, i.e. Flora Europaea (Tutin et al. 1968, Tutin et al. 1972, Tutin et al. 1976, Tutin et al. 1980, Tutin et al. 1993), Flora Hellenica (Strid and Tan 1997, Strid and Tan 2002), Mountain Flora of Greece (Strid 1986, Strid and Tan 1991), Flora of Turkey and the East Aegean Islands (Davis 1965, Davis 1967, Davis 1970, Davis 1972, Davis 1975, Davis 1978, Davis 1982, Davis 1984) and Atlas of the Aegean Flora (Strid 2016a, Strid 2016b). Additional, more specific literature was used for certain taxa, as reported. Taxonomic nomenclature mainly follows Dimopoulos et al. (2013) and Dimopoulos et al. (2016), except for those taxa reported in Greece for the first time or when otherwise stated. For the type specimens consulted and the specimens used as an identification and distribution aid, we report collector and collection number and/or accession number (a.n.) in the text, together with the Herbarium acronym, in accordance with Thiers (2018) (continuously updated). The plant descriptions and measurements are based on the collected samples and may represent only part of the morphological variation encountered in each taxon. A species or subspecies is characterised as endemic when it is confined to the political borders of a single country, in our case Greece, unless if defined in a different way in the text. A range-restricted taxon occupies a limited area of distribution not exceeding 500 km or, in other words, its most distant known populations are separated by a linear distance of 500 km or less. The phytogeographical regions of Greece can be found in Strid and Tan (1997). All plant specimens reported in this work have been deposited in the Herbarium of Biology Department, National and Kapodistrian University of Athens (ATHU). All photographs were taken in the field by the authors, as indicated in the legends. The taxa are presented in alphabetical order according to families and within families according to genera.

## Taxon treatments

### Anthericum ramosum

L.

670C062B-2777-5AC1-8A1C-3C10E5FD2663

Anthericum ramosum L. in Sp. Pl. 1: 310 (1753)

#### Materials

- **Type status:** Other material. **Occurrence:** recordedBy: K. Goula & N.A. Katsaros; **Taxon:** scientificName: *Anthericumramosum*; family: Anthericaceae; genus: Anthericum; specificEpithet: *ramosum*; taxonRank: species; **Location:** continent: Europe; country: Greece; stateProvince: Nomos Dramas; verbatimLocality: Mt. Orvilos, 2 km NE of Katafito Village; verbatimElevation: 860 m; verbatimLatitude: 41°21′; verbatimLongitude: 23°42′; **Identification:** identifiedBy: K. Goula & Th. Constantinidis; **Event:** eventDate: 4 August 2021; habitat: rocky, calcareous slope; **Record Level:** collectionID: 3230; institutionCode: ATHU; basisOfRecord: Specimen

#### Taxon discussion

**Figure 1.**
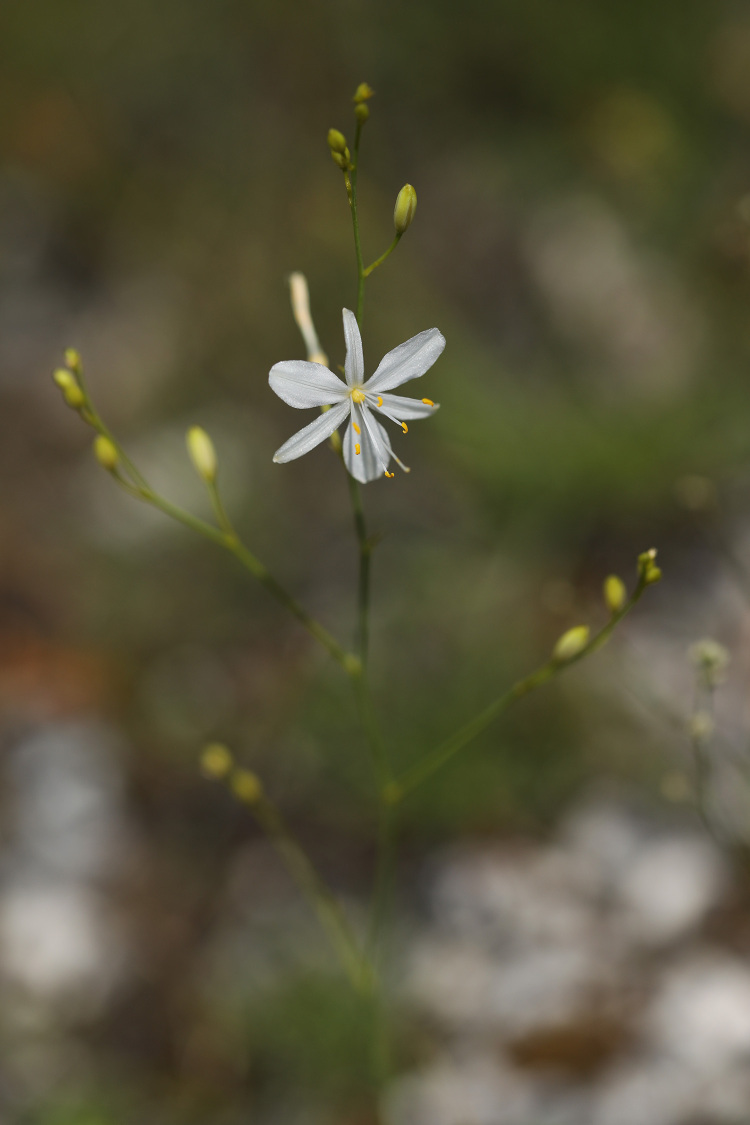
*Anthericumramosum* from Mt Orvilos (photo: K. Goula).

A new record for the Greek flora. *Anthericumramosum*, the generitype of *Anthericum* L., has a predominantly central European distribution that reaches parts of Russia and Asiatic Turkey to the East (Webb 1980, Matthews 1984). Although reported “*in Europae australioris*” by Linnaeus (1762) (type: Herb. Linn. no. 432.6, LINN!, see Jarvis et al. 1993) it has not been reported in Greece so far. Our specimen from the foothills of Mt. Orvilos (Fig. 1), at a linear distance of ca. 5.5 km south of the Greek-Bulgarian border, has a paniculate inflorescence with 2-6, 6.5-11 cm long branches, white tepals 9-12 mm long, a straight style and an almost spherical, obtuse capsule 5-5.5 x 4.5-6 mm with a short remnant of the style base. It differs considerably from *A.liliago* L., which is widespread in Greece, being reported from the south mainland (north Peloponnisos) to the northern borders (Andersson 1991a). The latter has a simple or sparingly branched inflorescence, larger tepals (13-20 mm), a somewhat curved style and a longer (8-11 x 6-7 mm) capsule with an acute apex.

### Trachomitum venetum

(L.) Woodson

A523D502-C225-5706-B1F0-E9CC182D4FB9

Trachomitum venetum (L.) Woodson in Ann. Miss. Bot. Gard. 17: 158 (1930)

#### Materials

- **Type status:** Other material. **Occurrence:** recordedBy: P. Doumas; **Taxon:** scientificName: *Trachomitumvenetum*; family: Apocynaceae; genus: Trachomitum; specificEpithet: *venetum*; taxonRank: species; **Location:** continent: Europe; country: Greece; stateProvince: Nomos Xanthis; verbatimLocality: Mandra beach, next to a drainage ditch; verbatimElevation: 0 m; verbatimLatitude: 40°57′; verbatimLongitude: 25°00′; **Identification:** identifiedBy: P. Doumas, K. Goula & Th. Constantinidis; **Event:** eventDate: 5 June 2021; habitat: on sandy shore; **Record Level:** collectionID: 42; institutionCode: ATHU; basisOfRecord: Specimen

#### Taxon discussion

**Figure 2.**
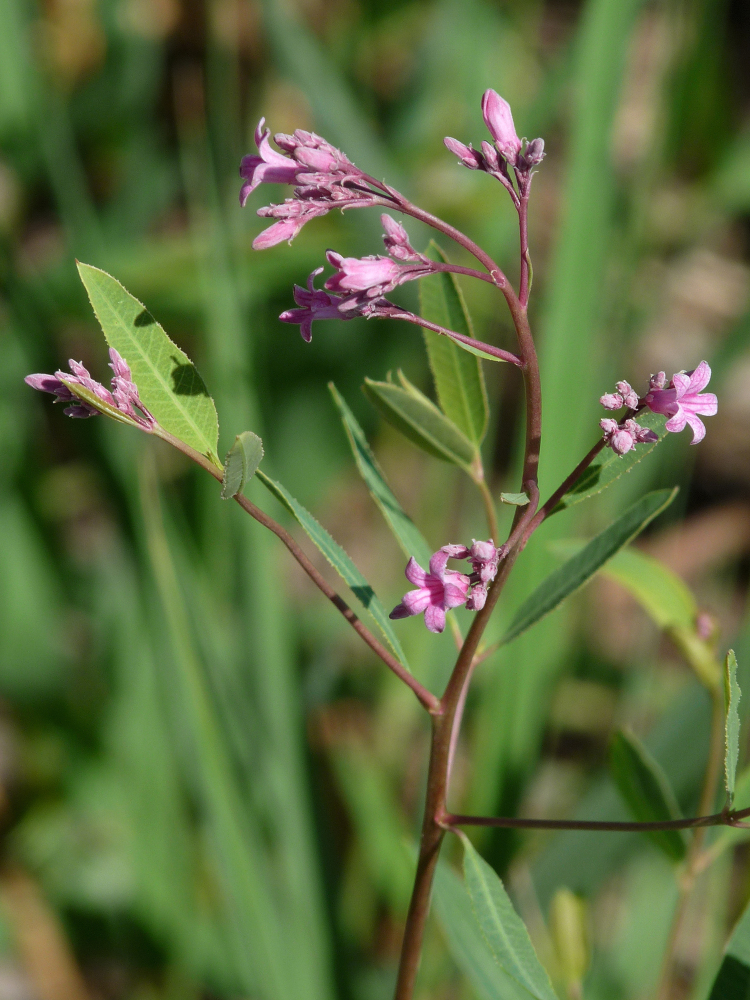
*Trachomitumvenetum* from Nomos Xanthis (photo: P. Doumas).

**Figure 3.**
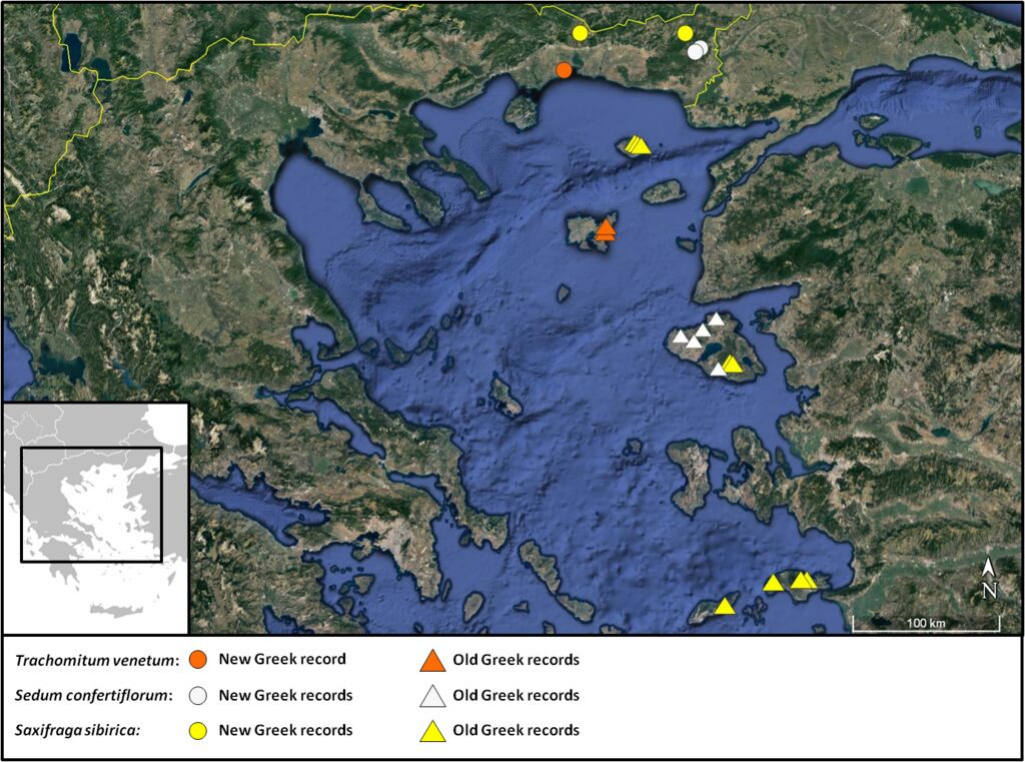
Distribution map of *Trachomitumvenetum*, *Sedumconfertiflorum* and *Saxifragasibirica* in Greece.

This is the second record of the taxonomically difficult genus *Trachomitum* in Greece (Fig. 2). The first one comes from the Island of Limnos, in the northern Aegean (Yannitsaros et al. 2001, Panitsa et al. 2003, Bergmeier et al. 2021) (Fig. 3). Having a different opinion than that of Stearn (1978) and the preceded authors, we attribute the plants from Nomos Xanthis and Limnos Island (*J. Krause & M. Ristow 265/19*, Limnos, Herb. Ristow!) to *Tr.venetum* (Tr.venetumsubsp.venetum) instead of *T.sarmatiense* Woodson (Tr.venetumsubsp.sarmatiense (Woodson) Avetisjan). This is in accordance with Rechinger (1974), who treated the genus in the same way for Flora Iranica, Woodson (1930) and Markgraf (1972). The Greek plants mostly have lanceolate, acute leaves, conspicuous bracts, lanceolate and acute calyx-lobes up to 2 mm and corolla-lobes 1/2 to almost as long as the corolla tube. Α comparison of Greek plants with the type specimen of *T.sarmatiense* (a.n. 00093153, GH!) showed significant differences in the above-mentioned characters. The latter has ovate to ovate-lanceolate leaves with obtuse apex, inconspicuous bracts, ovate and obtuse calyx-lobes up to 1 mm and corolla-lobes shorter than 1/3 of the corolla tube. Furthermore, *T.sarmatiense* appears to have a more eastern distribution (Woodson 1930, Pobedimova 1952, Markgraf 1972, Rechinger 1974, Euro+Med 2006+ [continuously updated]), whereas *T.venetum* grows in south-east Europe and the eastern Mediterranean area, including Cyprus. As a consequence, *Tr.sarmatiense* should be excluded from the Greek flora.

### Asphodeline taurica

(Pall.) Endl.

E69591DA-178D-5A93-A077-75F908BC373D

Asphodeline taurica (Pall.) Endl. in Cat. Horti Vindob. 1: 142 (1842)

#### Materials

- **Type status:** Other material. **Occurrence:** recordedBy: K. Goula; **Taxon:** scientificName: *Asphodelinetaurica*; family: Asphodelaceae; genus: Asphodeline; specificEpithet: *taurica*; taxonRank: species; **Location:** continent: Europe; country: Greece; stateProvince: Nomos Dramas; verbatimLocality: Mt. Orvilos, ca. 1 km SE of the main summit; verbatimElevation: 1760 m; verbatimLatitude: 41°22′; verbatimLongitude: 23°73′; **Identification:** identifiedBy: K. Goula; **Event:** eventDate: 29 May 2016; habitat: rocky, calcareous slope; **Record Level:** basisOfRecord: Photograph
- **Type status:** Other material. **Occurrence:** recordedBy: K. Goula, U. Gerhard & M. Vlachos; **Taxon:** scientificName: Asphodelinetaurica; family: Asphodelaceae; genus: Asphodeline; specificEpithet: taurica; taxonRank: species; **Location:** continent: Europe; country: Greece; stateProvince: Nomos Kavalas; verbatimLocality: Mt. Pangeo, above abandoned forest settlement; verbatimElevation: 1290 m; verbatimLatitude: 40°54′; verbatimLongitude: 24°08′; **Identification:** identifiedBy: K. Goula; **Event:** eventDate: 4 June 2019; habitat: rocky, calcareous slope; **Record Level:** basisOfRecord: Photograph
- **Type status:** Other material. **Occurrence:** recordedBy: P. Doumas; **Taxon:** scientificName: Asphodelinetaurica; family: Asphodelaceae; genus: Asphodeline; specificEpithet: taurica; taxonRank: species; **Location:** continent: Europe; country: Greece; stateProvince: Nomos Xanthis; verbatimLocality: Mt. Achladovouno, 350 m NE of the main summit; verbatimElevation: 1300 m; verbatimLatitude: 41°10′; verbatimLongitude: 24°48′; **Identification:** identifiedBy: P. Doumas & K. Goula; **Event:** eventDate: 16 May 2021; habitat: rocky, calcareous slope; **Record Level:** collectionID: 23; institutionCode: ATHU; basisOfRecord: Specimen

#### Taxon discussion

**Figure 4.**
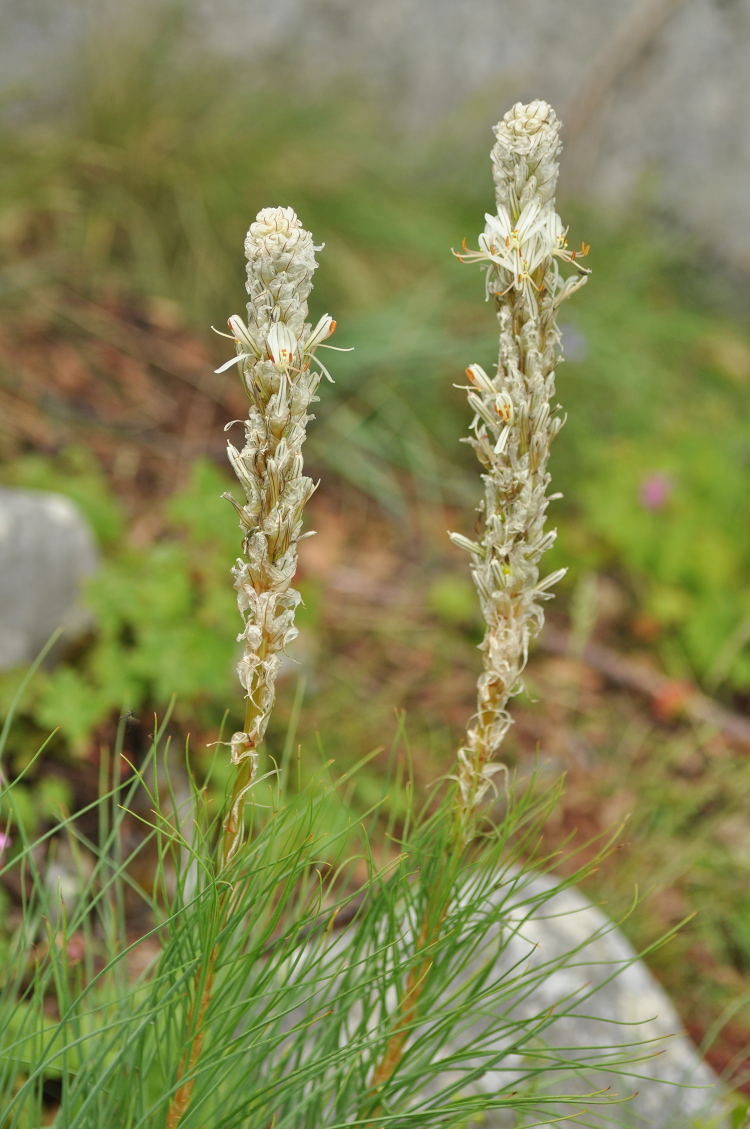
*Asphodelinetaurica* from Mt. Pangeo (photo: K. Goula).

Scattered in the mountains of north and central Greece, *Asphodelinetaurica* reaches Sterea Ellas and north Peloponnisos to the south, with its southernmost Greek locality on Mt. Chelmos (Goulimis 1960, Andersson 1991b, Authier 1994, Authier 2002, Tan et al. 2006, Strid et al. 2020; herbarium specimens preserved in B with JACQ-IDs 655330 and 655358). In the floristic region of NE Greece, the species has been reported from Mt. Falakro (Tan et al. 2006), Mt. Papikio (Andersson 1991b) and Mt. Angistro (JACQ-ID 655358, B!). Our records (Fig. 4) add three new mountains to the distribution of this species, uncommon in Greece.

### Aster amellus

L.

6D1B08ED-2E2E-5983-A6FC-D7A295BDD710

Aster amellus ***Asteramellus* L.**

#### Materials

- **Type status:** Other material. **Occurrence:** recordedBy: P. Doumas; **Taxon:** scientificName: Asteramellus; family: Asteraceae; genus: Aster; specificEpithet: amellus; taxonRank: species; **Location:** continent: Europe; country: Greece; stateProvince: Nomos Dramas; verbatimLocality: Mt. Rodopi, ca. 1 km NW of Trachoni settlement; verbatimElevation: 753 m; verbatimLatitude: 41°21′; verbatimLongitude: 24°37′; **Identification:** identifiedBy: P. Doumas, K. Goula & Th. Constantinidis; **Event:** eventDate: 12 September 2021; habitat: Quercus sp. forest, waysides, margins and clearings; **Record Level:** collectionID: 59; institutionCode: ATHU; basisOfRecord: Specimen

#### Taxon discussion

This is a new addition to the Greek flora. Previous old reports are considered erroneous.

The *Asteramellus* records in Greece have a long history. Old reports from the dry habitats of Attiki near Athens under either *A.amellus*, as in Boissier (1875) (*hab. in collibus apricis Atticae*) or *A.atticus* Pall. (Tournefort 1700, Jacquin 1778, Sibthorp and Smith 1813) were soon disputed. Halácsy (1902) noted that these old records were probably erroneous; his opinion is followed in the contemporary literature (see Dimopoulos et al. 2013). Fraas (1845), however, referred to *A.amellus* as a plant of salt marshes and coastal swamps from the area of Faliro, south-west of the Athens centre and used the common name “*valtokratis*” (from the Greek words *βάλτος*, i.e. marsh and *κράτος*, i.e. power, strength) to identify it. Such a habitat is alien to *A.amellus*, a species of drier localities that colonises grasslands, slopes, waysides, scrub formations and wood margins and clearings (Merxmüller and Schreiber 1976). It is quite safe to assume that Fraas (1845), when mentioning *A.amellus*, was actually referring to *Tripoliumpannonicum* (Jacq.) Dobrocz. (*Astertripolium* L.), a plant known for its preference for marshes and brackish waters and still found in Attiki. Due to lack of any further evidence during the last 150 years, *A.amellus* was justifiably excluded from the Greek flora.

**Figure 5.**
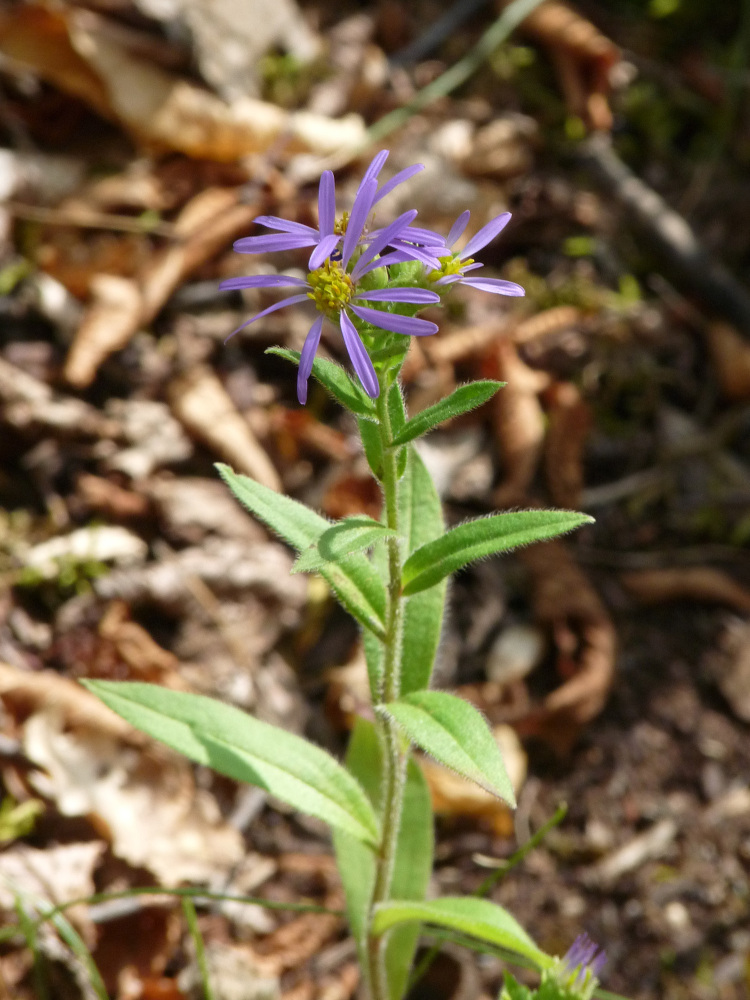
*Asteramellus* from Mt. Rodopi (photo: P. Doumas).

Our collection of *Asteramellus* from the Rodopi area (Fig. 5) is the first that confirms this species as an indigenous member of the Greek flora. Its collection locality lies at a linear distance of ca. 8.5 km south of the Greek-Bulgarian border. According to the distribution map provided by Münzbergová et al. (2011), the Greek locality is marginally outside the total distribution range of the species. It is implied that *A.amellus* was suspiciously absent from the Greek territory. From a morphological point of view, the species is polymorphic and includes three different cytotypes: diploids (2n=2x=18), tetraploids (2n=4x=36) and hexaploids (2n=6x=54). Certain literature sources and taxonomic databases recognise subspecific entities that are mostly based on the treatment of Tamamshyan (1959). The Greek samples are pubescent on stems and mostly have scabrid leaves, bear numerous small capitula 9-11 mm wide (ligules excluded), with green, subacute and ciliate involucral bracts often pubescent dorsally. They come closer to subsp. bessarabicus (Rchb.) Soó, but the whole group needs further investigation.

### Centaurea marmorea

Bornm. & Soska

9F5F5EBF-9484-5135-AE37-D391B43278E6

Centaurea marmorea Bornm. & Soska in Repert. Spec. Nov. Regni Veg. 42: 127 (1937)

#### Materials

- **Type status:** Other material. **Occurrence:** recordedBy: K. Goula & N.A. Katsaros; **Taxon:** scientificName: Centaureamarmorea; family: Asteraceae; genus: Centaurea; specificEpithet: marmorea; taxonRank: species; **Location:** continent: Europe; country: Greece; stateProvince: Nomos Dramas; verbatimLocality: Mt. Falakro; verbatimElevation: 1750 m; verbatimLatitude: 40°54′; verbatimLongitude: 24°08′; **Identification:** identifiedBy: K. Goula & Th. Constantinidis; **Event:** eventDate: 3 August 2021; habitat: rocky slopes and road embankments, on marbles; **Record Level:** collectionID: 3229; institutionCode: ATHU; basisOfRecord: Specimen

#### Taxon discussion

**Figure 7.**
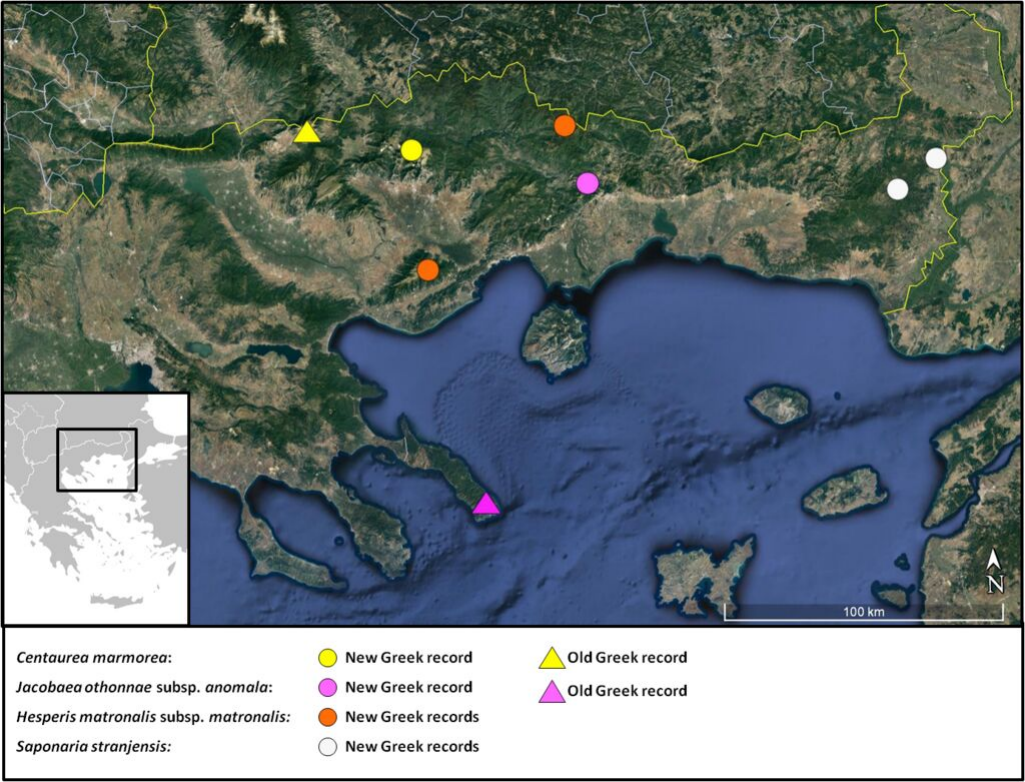
Distribution map of *Centaureamarmorea*, Jacobaeaothonnaesubsp.anomala, Hesperismatronalissubsp.matronalis and *Saponariastranjensis* in Greece.

A rare, local and range-restricted Balkan endemic shared between North Macedonia and Greece. It has been recorded with certainty only from the Prilep area (North Macedonia) and Mt. Orvilos (NE Greece, see Gamal-Eldin and Wagenitz 1991). The new locality on Falakro (Fig. 6) adds a new mountain locality to the species’ distribution, which is thus extended to the east (Fig. 7).

### Jacobaea othonnae anomala

(Sm.) Sutorý

8092AC64-A118-5AD8-806D-C16A693B129D

Jacobaea othonnae subsp. anomala (Sm.) Sutorý in Phytotaxa 510(1): 65 (2021)

#### Materials

- **Type status:** Other material. **Occurrence:** recordedBy: P. Doumas; **Taxon:** scientificName: Jacobaeaothonnae subsp. anomala; family: Asteraceae; genus: Jacobaea; specificEpithet: othonnae; infraspecificEpithet: anomala; taxonRank: subspecies; **Location:** continent: Europe; country: Greece; stateProvince: Nomos Xanthis; verbatimLocality: Mt. Achladovouno, NE of the main summit; verbatimElevation: 1154 m; verbatimLatitude: 41°10′; verbatimLongitude: 24°48′; **Identification:** identifiedBy: P. Doumas, K. Goula & Th. Constantinidis; **Event:** eventDate: 9 July 2020; habitat: Fagus sylvatica forest, next to a spring; **Record Level:** collectionID: s.n.; institutionCode: ATHU; basisOfRecord: Specimen

#### Taxon discussion

**Figure 8.**
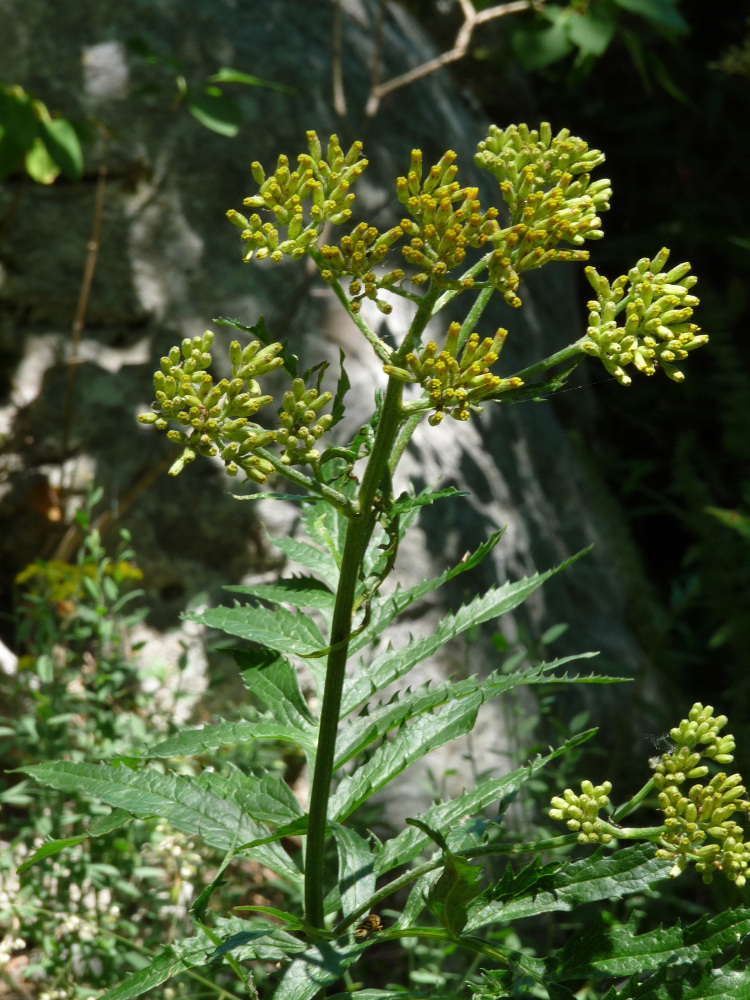
Jacobaeaothonnaesubsp.anomala from Mt. Achladovouno (photo: P. Doumas).

*Jacobaeaothonnae* is rare in Greece, known from the floristic regions of North Pindos (NPi) and NE (Dimopoulos et al. 2013). In North Pindos, specifically, there is only one record from Mt. Timfi (Authier 2014), while in the NE, there are records from Mt. Athos (Boissier 1875, Authier 2014), Mt. Pangeo (a.n. 1983136, LD!) and Mt. Vrondous (Authier 2014). According to Sutorý (2021), the Mt. Athos plants are somewhat isolated, have woolly-tomentose synflorescences and involucres and belong to the distinct and local subsp. anomala (Sm.) Sutorý. The rest of the north-eastern populations are attributed to the typical subsp. othonnae and have glabrous synflorescences and involucres. The plants of Mt. Achladovouno (Fig. 8) seem to break this distribution pattern, as they present woolly-tomentose synflorescences, particularly at young age and, therefore, fit subsp. anomala. Our new collection adds a second mountain locality to subsp. anomala and extends its distribution ca. 120 km to the north-east (Fig. 7).

### Takhtajaniantha austriaca

(Willd.) Zaika, Sukhor. & N.Kilian

5F254FBD-E1A6-5529-AC7E-9EB4078B06FE

Takhtajaniantha austriaca (Willd.) Zaika, Sukhor. & N.Kilian in PhytoKeys 137: 72 (2020) [*Scorzoneraaustriaca* Willd.]

#### Materials

- **Type status:** Other material. **Occurrence:** recordedBy: P. Doumas; **Taxon:** scientificName: Takhtajanianthaaustriaca; family: Asteraceae; genus: Takhtajaniantha; specificEpithet: austriaca; taxonRank: species; **Location:** continent: Europe; country: Greece; stateProvince: Nomos Xanthis; verbatimLocality: Mt. Achladovouno, just below the summit ridge; verbatimElevation: 1332 m; verbatimLatitude: 41°10′; verbatimLongitude: 24°47′; **Identification:** identifiedBy: P. Doumas, K. Goula & Th. Constantinidis; **Event:** eventDate: 16 May 2021; habitat: rocky, calcareous slopes; **Record Level:** collectionID: 29; institutionCode: ATHU; basisOfRecord: Specimen

#### Taxon discussion

**Figure 9.**
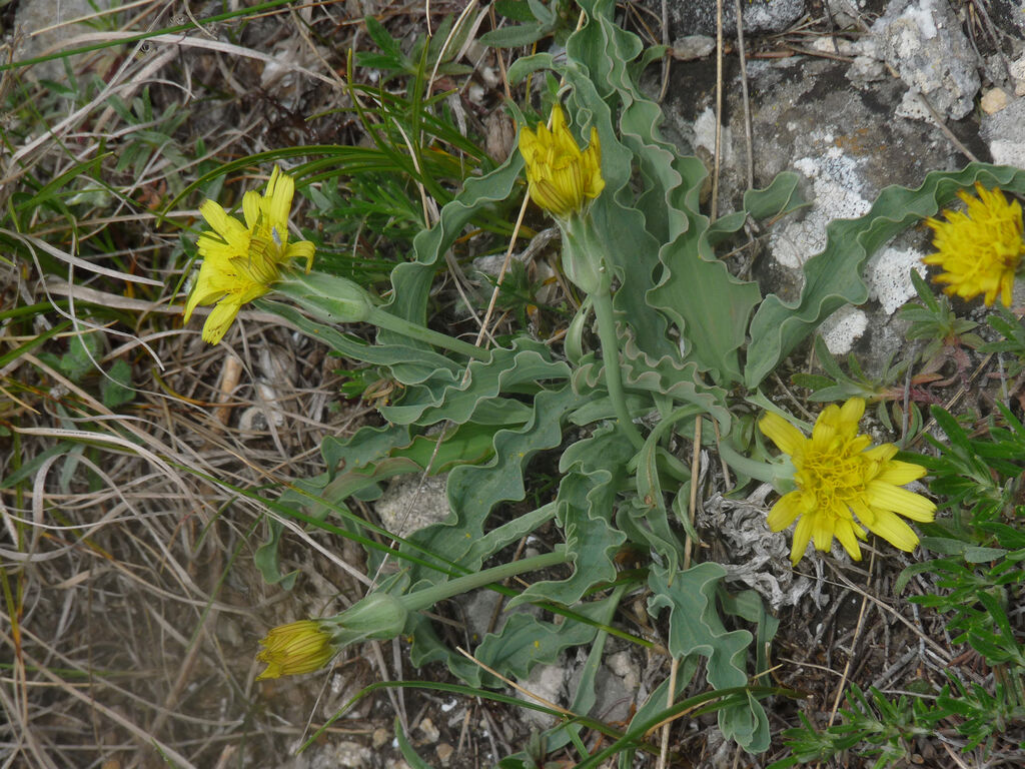
*Takhtajanianthaaustriaca* from Mt. Achladovouno (photo: P. Doumas).

A Euro-Siberian floristic element reported twice in Greece: on the high-altitude serpentine rocks of Mt. Smolikas (Lack and Kilian 1991) and on the northern foothills of Mt. Olimbos (Strid and Franzén 1984). Lack and Kilian (1991) recorded the species as *Scorzoneraaustriaca* and noted that the Mt. Smolikas population is somehow intermediate between subsp. austriaca and subsp. bupleurifolia (Pouzolz) Bonnier. The Mt. Achladovouno population (Fig. 9) is new for the NE region and comes closer to the typical subspecies because of its basal leaves with lamina gradually narrowed into the petiole (Chater 1976). Nomenclature follows Zaika et al. (2020), who transferred *Scorzoneraaustriaca* to *Takhtajaniantha*, in accordance with carpological, anatomical and molecular phylogenetic evidence.

### Onosma kittanae

Strid ex Stefanović, Kit Tan & Iatroú

4806541A-70F9-590E-B9DA-53BBA125F533

Onosma kittanae Strid ex Stefanović, Kit Tan & Iatroú in Pl. Syst. Evol. 242(1-4): 157 (2003)

#### Materials

- **Type status:** Other material. **Occurrence:** recordedBy: P. Doumas; **Taxon:** scientificName: Onosmakittanae; family: Boraginaceae; genus: Onosma; specificEpithet: kittanae; taxonRank: species; **Location:** continent: Europe; country: Greece; stateProvince: Nomos Xanthis; verbatimLocality: ca. 2.4 km SE of Potamochori Village; verbatimElevation: 553 m; verbatimLatitude: 41°14′; verbatimLongitude: 25°04′; **Identification:** identifiedBy: P. Doumas & K. Goula; **Event:** eventDate: 15 May 2021; habitat: scrub, on serpentine; **Record Level:** collectionID: 19; institutionCode: ATHU; basisOfRecord: Specimen

#### Taxon discussion

**Figure 10.**
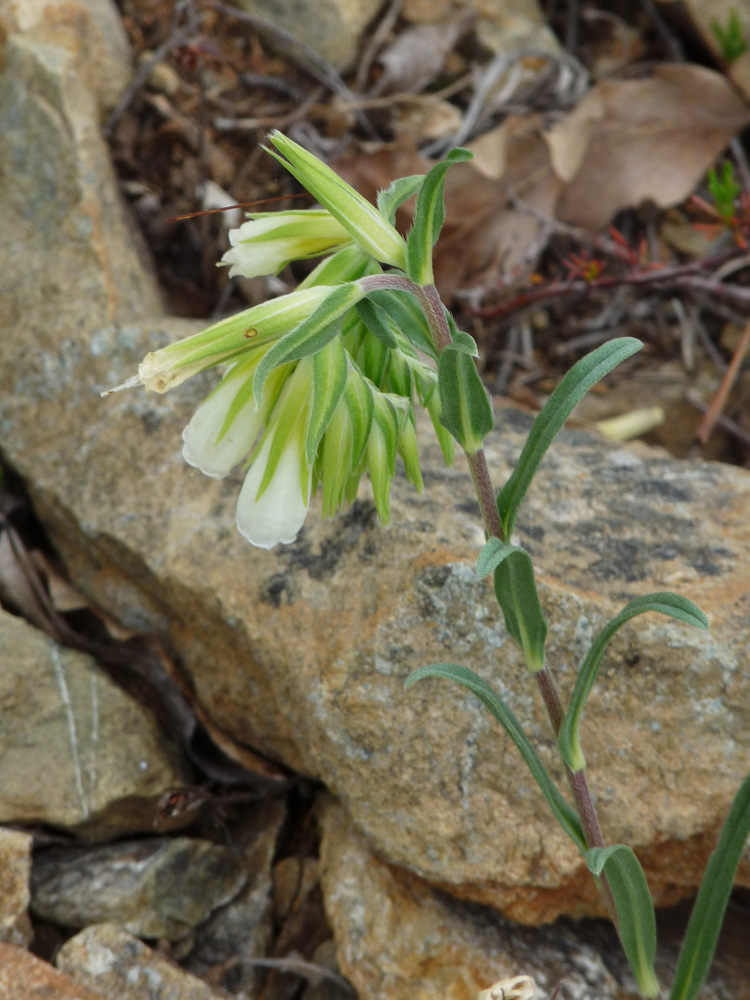
*Onosmakittanae* from Nomos Xanthis (photo: P. Doumas).

**Figure 11.**
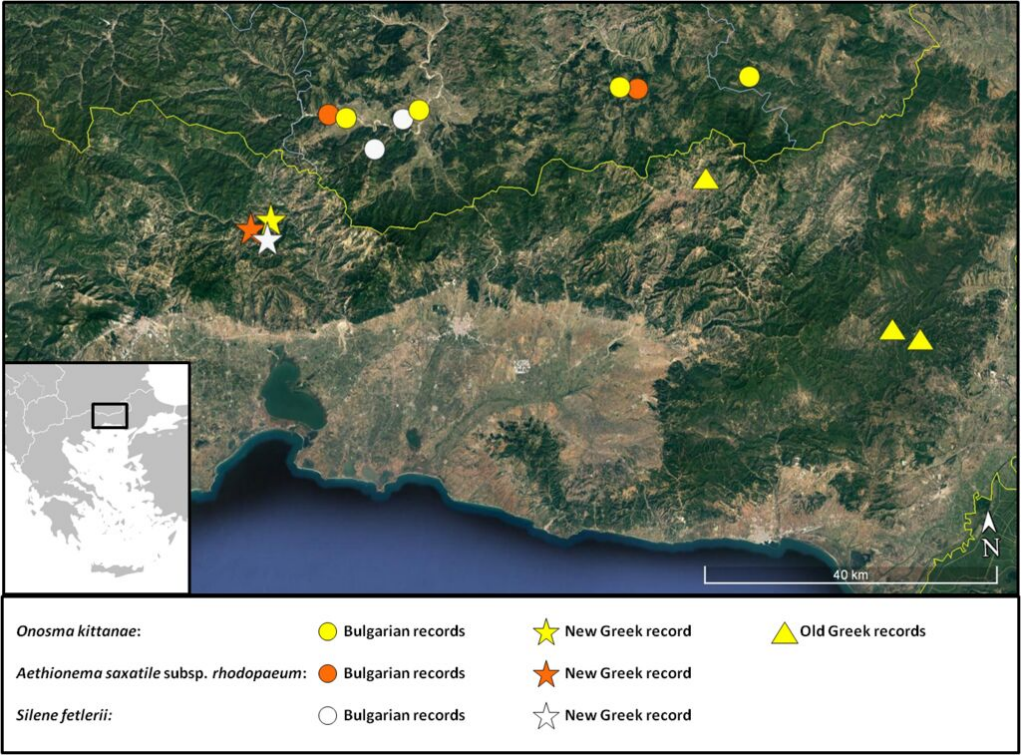
Distribution map of *Onosmakittanae*, Aethionemasaxatilesubsp.rhodopaeum and *Silenefetlerii* in Greece and Bulgaria.

*Onosmakittanae* was initially described as endemic to Greece (Stevanović et al. 2003), until Teppner and Karl (2017) pointed out its similarities to *O.pavlovae* Petrova & Kit Tan (*O.bulgarica* D. Pavlova non Velenovský) from Bulgaria and treated the latter as a synonym of *O.kittanae*. The species remains a rare, range-restricted Balkan endemic confined to the serpentine substrates of south Bulgaria and NE Greece. In Greece, only two localities were known so far: around the *locus classicus* in Nomos Evrou (Stevanović et al. 2003, Teppner and Karl 2017) and between Organi and Chloi Villages of Nomos Rodopis (Strid 2018). Our third, new locality (Fig. 10) forms the westernmost distribution border for the species (Fig. 11). According to the [B1ab(ii iii iv) + 2ab(ii iii iv)] criteria, *O.kittanae* fits the Endangered Category at national level (IUCN 2012).

### Aethionema saxatile rhodopaeum

(D.K. Pavlova) Constantinidis, Kougioumoutzis & Kalpoutzakis 2017

8080E6BA-C421-57D6-BF78-49CB586056DB

Aethionema saxatile subsp. rhodopaeum (D.K. Pavlova) Constantinidis, Kougioumoutzis & Kalpoutzakis in Plant Biosyst. 151 (1): 115 (2017)

#### Materials

- **Type status:** Other material. **Occurrence:** recordedBy: P. Doumas; **Taxon:** scientificName: Aethionemasaxatile subsp. rhodopaeum; family: Brassicaceae; genus: Aethionema; specificEpithet: saxatile; infraspecificEpithet: rhodopaeum; taxonRank: subspecies; **Location:** continent: Europe; country: Greece; stateProvince: Nomos Xanthis; verbatimLocality: between the Villages of Potamochori and Korifi, close to Kompsatos river belt; verbatimElevation: 550 m; verbatimLatitude: 41°14′; verbatimLongitude: 25°04′; **Identification:** identifiedBy: P. Doumas & Th. Constantinidis; **Event:** eventDate: 27 March 2019; habitat: rocky, serpentine slope with scrub, in deciduous Quercus forest; **Record Level:** collectionID: s.n.; institutionCode: ATHU; basisOfRecord: Specimen

#### Taxon discussion

A new record for the Greek flora.

As currently understood, *Aethionemasaxatile* (L.) R.Br. is quite variable in Greece. It consists of four subspecies with a more or less distinct geographical distribution that deserve the rank of subspecies (Andersson et al. 1983, Kougioumoutzis et al. 2017). Certain specimens from NW Greece resemble a fifth subspecies, subsp. saxatile, but statistical analyses of morphological characters showed that they cannot be placed with certainty under either subsp. oreophilum or subsp. saxatile. A related species, *Ae.rhodopaeum* D.K. Pavlova (Pavlova 2007), so far considered endemic to Bulgaria, was treated as another subspecies of the *Ae.saxatile* group by Kougioumoutzis et al. (2017). It is a serpentine endemic with a local distribution to suitable geological substates of the Rodopi area.

The cited specimen, collected in Greece in 2019 (Fig. 12), has a combination of features that place it under Ae.saxatilesubsp.rhodopaeum: a) short stems, usually less than 10 cm long; b) unilocular fruits present at a relatively high proportion (>30-40% of total fruit number); c) lack of appendages on filaments; d) dark pink to pink-purplish petals and e) a particular preference for the rocky, serpentine habitats of the Kompsatos River valley.

The following key helps in distinguishing all *Αethionema saxatile* taxa found in Greece, Table 1.

Following a morphological and statistical approach, the close taxonomic proximity of subsp. rhodopaeum to the rest of the *Ae.saxatile* taxa is unquestionable. However, a recent phylogenetic reconstruction places it away from *Ae.saxatile* as it forms a monophyletic group with the annual Asiatic *Ae.syriacum* (Boiss.) Bornm. and the perennial *Ae.orbiculatum* (Boiss.) Hayek, a local endemic of Mt. Athos, Greece (Mohammadin et al. 2017). A more detailed investigation of the whole group may be necessary in the future.

subsp. rhodopaeum was previously known from a few, small populations in Bulgaria, where it has been assessed as Endangered, according to the IUCN Categories and Criteria (Pavlova 2007). The Greek population was found at a distance of 11 km south of the Greek-Bulgarian border, consists of a few dozens of plants and covers an area of just a few square metres (Fig. 11). Its discovery expands the extent of occurrence, area of occupancy and population size of this rare plant; however, it remains an Endangered species at global level, according to Criteria [B1ab(ii iv) + 2ab(ii iv)]. At national level, the single Greek population fits the Category of Critically Endangered [B1ab(i ii iii iv) + 2ab(i ii iii iv)] (IUCN 2012).

### Hesperis matronalis matronalis

L. 1753

582884DC-4326-5665-8BF4-DDEE5A00FA5C

Hesperis matronalis L. subsp. matronalis in Sp. Pl. 663 (1753)

#### Materials

- **Type status:** Other material. **Occurrence:** recordedBy: P. Doumas; **Taxon:** scientificName: Hesperismatronalis subsp. matronalis; family: Brassicaceae; genus: Hesperis; specificEpithet: matronalis; infraspecificEpithet: matronalis; taxonRank: subspecies; **Location:** continent: Europe; country: Greece; stateProvince: Nomos Dramas; verbatimLocality: Mt. Rodopi, area of Arkoudorema, ca. 3.6 km NNE of Erimanthos forest village; verbatimElevation: 1194 m; verbatimLatitude: 41°21′; verbatimLongitude: 24°43′; **Identification:** identifiedBy: P. Doumas, K. Goula & Th. Constantinidis; **Event:** eventDate: 27 June 2021; habitat: Fagus sylvatica forest, next to a stream; **Record Level:** collectionID: 44; institutionCode: ATHU; basisOfRecord: Specimen

#### Taxon discussion

Α new subspecies for the Greek flora. *Hesperismatronalis* is a widespread and variable species divided into 6 or 7 subspecies, of which subsp. cladotricha (Borbás) Hayek has been recorded as a rare plant of the Mt. Timfi area in north-west Greece (Goulimis 1960, Ganiatsas 1971, Authier 1993, Bergmeier 2008). Our collection from NE Greece (Fig. 13) presents a monomorphic indumentum on upper and lower leaf surfaces consisting of simple eglandular hairs; branched hairs are very scarce and rarely found along veins and margins. It clearly fits subsp. matronalis because of its unbranched hairs, pink-purple flowers and petiolate middle and upper stem leaves. In contrast, a specimen of subsp. cladotricha examined (*Κ. Goula & Ν.Α. Katsaros 3036*, Mt Timfi, Skamneli, ATHU!) presents an indumentum of predominantly bifurcate (and occasionally trifurcate) eglandular hairs; simple hairs are very rare or absent. *Hesperismatronalis* is present in all the floristic regions of neighbouring Bulgaria (Assyov and Petrova 2012) and, therefore, its occurrence in NE Greece was to be expected. The Greek population grows in a very natural habitat away of any human interference and is clearly indigenous. Interestingly, a Greek collection of subsp. matronalis already appeared amongst the specimens examined by Duran and Ocak (2005) from Mt. Pangeo (Pangaeo, west of Kawala, *Fagus* forest, 3500 ft., *J.D.A. Stainton* 7737, 21.06.1959, K), but this record has passed unnoticed (Fig. 7).

### Campanula orphanidea

Boiss.

949C0795-70B2-5C9E-80B3-0BD2E108804D

Campanula orphanidea Boiss. in Fl. Orient. 3: 897 (1875)

#### Materials

- **Type status:** Other material. **Occurrence:** recordedBy: P. Doumas; **Taxon:** scientificName: Campanulaorphanidea; family: Campanulaceae; genus: Campanula; specificEpithet: orphanidea; taxonRank: species; **Location:** continent: Europe; country: Greece; stateProvince: Nomos Xanthis; verbatimLocality: Mt. Achladovouno, the summit area; verbatimElevation: 1395 m; verbatimLatitude: 41°10′; verbatimLongitude: 24°48′; **Identification:** identifiedBy: P. Doumas & K. Goula; **Event:** eventDate: 4 August 2021; habitat: rocky, calcareous slopes; **Record Level:** collectionID: 48; institutionCode: ATHU; basisOfRecord: Specimen

#### Taxon discussion

A local and range-restricted species shared between NE Greece and south Bulgaria. In Greece, it has been reported from the mountains of Athos, Pangeo, Orvilos, Menikio and Falakro, all belonging to the NE floristic region (Hartvig 1991). The new locality on Mt. Achladovouno extends the distribution range of the species to the east.

### Cerastium decalvans orbelicum

(Velen.) Stoj. & Stef. 1948

C96FE15B-97FB-5826-BC42-789378EE528A

Cerastium decalvans subsp. orbelicum (Velen.) Stoj. & Stef. in Fl. Bulg.: 416 (1948)

#### Materials

- **Type status:** Other material. **Occurrence:** recordedBy: P. Doumas; **Taxon:** scientificName: Cerastiumdecalvans subsp. orbelicum; family: Caryophyllaceae; genus: Cerastium; specificEpithet: decalvans; infraspecificEpithet: orbelicum; taxonRank: subspecies; **Location:** continent: Europe; country: Greece; stateProvince: Nomos Xanthis; verbatimLocality: Mt. Achladovouno, ca. 720 m NE of the main summit; verbatimElevation: 1170 m; verbatimLatitude: 41°10′; verbatimLongitude: 24°48′; **Identification:** identifiedBy: P. Doumas & K. Goula; **Event:** eventDate: 16 May 2021; habitat: rocky, calcareous slope; **Record Level:** collectionID: 24; institutionCode: ATHU; basisOfRecord: Specimen
- **Type status:** Other material. **Occurrence:** recordedBy: P. Doumas; **Taxon:** scientificName: Cerastiumdecalvans subsp. orbelicum; family: Caryophyllaceae; genus: Cerastium; specificEpithet: decalvans; infraspecificEpithet: orbelicum; taxonRank: subspecies; **Location:** continent: Europe; country: Greece; stateProvince: Nomos Xanthis; verbatimLocality: Mt. Achladovouno, 840 m NW of the main summit, just below the summit ridge; verbatimElevation: 1353 m; verbatimLatitude: 41°10′; verbatimLongitude: 24°47′; **Identification:** identifiedBy: P. Doumas & K. Goula; **Event:** eventDate: 4 July 2021; habitat: rocky, calcareous slope; **Record Level:** collectionID: 53; institutionCode: ATHU; basisOfRecord: Specimen

#### Taxon discussion

A range-restricted subspecies, endemic to NE Greece, Bulgaria and North Macedonia (Dimopoulos et al. 2013). *Cerastiumdecalvans* has been reported from the mountains of Belles, Orvilos, Menikio, Pangeo, Falakro and Lekani in the floristic region of NE Greece (Strid 1986); however, the only records of subsp. orbelicum come from Mt. Belles and Mt. Menikio (Strid 1997a). The locality on Mt. Achladovouno is the easternmost border limit of the subspecies’ distribution in Greece.

### Dianthus petraeus orbelicus

(Velen.) Greuter & Burdet 1982

62554D61-4445-5C6D-A11E-418BF93DEB75

Dianthus petraeus subsp. orbelicus (Velen.) Greuter & Burdet in Willdenowia 12: 187 (1982)

#### Materials

- **Type status:** Other material. **Occurrence:** recordedBy: P. Doumas; **Taxon:** scientificName: Dianthus petraeus subsp. orbelicus; family: Caryophyllaceae; genus: Dianthus; specificEpithet: petraeus; infraspecificEpithet: orbelicus; taxonRank: subspecies; **Location:** continent: Europe; country: Greece; stateProvince: Nomos Xanthis; verbatimLocality: Mt. Achladovouno, ca. 700m NE of the main summit; verbatimElevation: 1165 m; verbatimLatitude: 41°10′; verbatimLongitude: 24°48′; **Identification:** identifiedBy: P. Doumas & K. Goula; **Event:** eventDate: 4 July 2021; habitat: rocky, calcareous slope; **Record Level:** collectionID: 50; institutionCode: ATHU; basisOfRecord: Specimen

#### Taxon discussion

This is a range-restricted subspecies, endemic to the eastern part of the Balkan Peninsula. In Greece, it is only known from the mountains of the NE floristic region: Athos, Belles, Pangeo, Menikio, Orvilos, Falakro and Rodopi (Strid 1997b). The new locality on Mt. Achladovouno extends the Greek distribution of the subspecies to the east.

### Petrorhagia cretica

(L.) P.W. Ball & Heywood

AF3EA239-166C-5A96-A42F-A83FF8184213

Petrorhagia cretica (L.) P.W. Ball & Heywood in Bull. Brit. Mus. (Nat. Hist.), Bot. 3: 142 (1964)

#### Materials

- **Type status:** Other material. **Occurrence:** recordedBy: K. Goula; **Taxon:** scientificName: Petrorhagiacretica; family: Caryophyllaceae; genus: Petrorhagia; specificEpithet: cretica; taxonRank: species; **Location:** continent: Europe; country: Greece; stateProvince: Nomos Evrou; verbatimLocality: ca. 5 km N of Avantas Village; verbatimElevation: 144 m; verbatimLatitude: 40°58′; verbatimLongitude: 25°54′; **Identification:** identifiedBy: K. Goula; **Event:** eventDate: 1 June 2019; habitat: rocky and gravelly places, on igneous substrates; **Record Level:** collectionID: 2881A; institutionCode: ATHU; basisOfRecord: Specimen

#### Taxon discussion

A very rare species in Greece, having been collected only three times from the floristic regions of North Central (NC), South Pindos (SPi) and Sterea Ellas (StE; Georgiou 1997, Pirini et al. 2010). Hayek (1927) reported it from Thraki (Thrace), but without any further information regarding its locality. Therefore, it is not known whether this reference should be attributed to the Greek, Turkish or Bulgarian part of the territory. The above-cited collection confirms the existence of the species in the Greek part of Thraki and consequently, adds a new floristic record to the NE region.

### Saponaria stranjensis

D. Jord.

5074E75F-6312-52DC-97D4-70742811D42F

Saponaria stranjensis D. Jord. in God. Sofiisk. Ubiv. Fiz.-Mat. Fak. 30: 400 (1933)

#### Materials

- **Type status:** Other material. **Occurrence:** recordedBy: P. Doumas; **Taxon:** scientificName: Saponariastranjensis; family: Caryophyllaceae; genus: Saponaria; specificEpithet: stranjensis; taxonRank: species; **Location:** continent: Europe; country: Greece; stateProvince: Nomos Evrou; verbatimLocality: W of Dadia Village, next to the road; verbatimElevation: 260 m; verbatimLatitude: 41°07′; verbatimLongitude: 26°07′; **Identification:** identifiedBy: P. Doumas & Th. Constantinidis; **Event:** eventDate: 4 July 2016; habitat: area surrounded by Pinus forest; **Record Level:** basisOfRecord: Photograph
- **Type status:** Other material. **Occurrence:** recordedBy: P. Doumas; **Taxon:** scientificName: Saponariastranjensis; family: Caryophyllaceae; genus: Saponaria; specificEpithet: stranjensis; taxonRank: species; **Location:** continent: Europe; country: Greece; stateProvince: Nomos Evrou; verbatimLocality: N of Soufli Town, next to the bank of Kamilopotamos stream; verbatimElevation: 34 m; verbatimLatitude: 41°12′; verbatimLongitude: 26°17′; **Identification:** identifiedBy: P. Doumas & Th. Constantinidis; **Event:** eventDate: 17 August 2019; habitat: Paliurus spina-christi thickets surrounded by mixed Pinus/Quercus forest; **Record Level:** collectionID: s.n.; institutionCode: ATHU; basisOfRecord: Specimen

#### Taxon discussion

**Figure 14.**
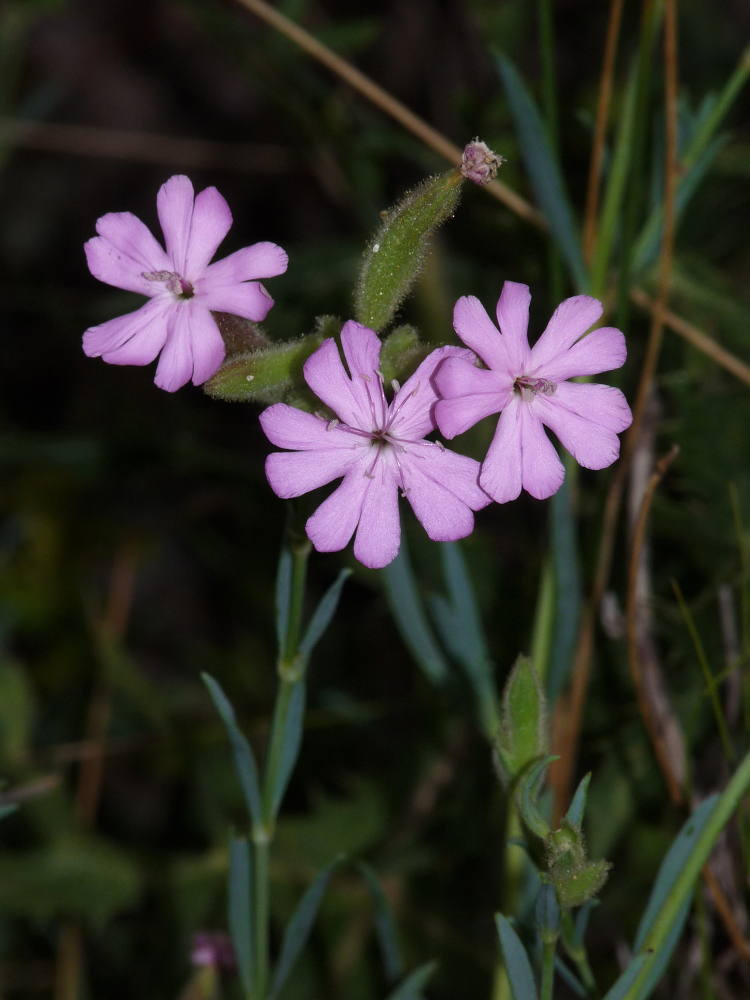
*Saponariastranjensis* from Nomos Evrou (photo: P. Doumas).

A new record for the Greek flora (Fig. 14). *Saponariastranjensis* is distributed in Bulgaria and European Turkey (Petrova and Vladimirov 2010). The two localities of Nomos Evrou extend the species’ known distribution to the south. Its closest taxonomic relative, *S.intermedia* Simmler, differs by usually having stems up to 50 cm (longer as a rule in *S.stranjensis*) and calyx with abundant eglandular hairs, rarely with a few short, glandular hairs, opposite to the calyx with abundant long and short glandular hairs in *S.stranjensis*. Although stem length may be a variable character and related to environmental conditions, our two randomly collected Greek specimens measure ca. 52 and 56 cm, respectively. Still, some authors consider those two taxa at subspecific rank rather than independent species (e.g. Jalas and Suominen 1988, Chater 1993), thus disagreeing with Mayer (1976).

In Greece, the two species have a clearly different distribution range: *S.intermedia* extends at the northern parts of Pindos Mountain chain and again found around Mt. Vourinos, at the NW parts of Greece (Phitos 1997), where it usually grows on serpentine. *S.stranjensis* occurs ca. 400 km to the east, near the small town of Soufli, close to the Greek-Turkish border. Τhe two species also present a different altitudinal range in Greece: *S.intermedia* grows at 850-1800 m a.s.l., whereas *S.stranjensis* has been found at 30-260 m a.s.l. In Bulgaria, the latter species has been recorded from 300 up to 500 m a.s.l. (Peev et al. 2015). The two Greek localities were found ca. 1.5 and 7.5 km west of the Greek-Turkish border, respectively (Fig. 7).

*Saponariastranjensis* has been assessed as Vulnerable in Bulgaria (Petrova and Vladimirov 2009, Peev et al. 2015) and is being protected by the National Biodiversity Act. Extinction has been observed in at least one previously known population in that country (Peev et al. 2015). In Greece, *S.stranjensis* is very local: its populations are geographically restricted and consist of a few dozens of mature plants only. We presume that the Greek populations are threatened, but a proper assessment according to the IUCN Categories and Criteria is still pending. The two Greek localities form a noteworthy extension of the nearest Bulgarian population: ca. 25 km to the SE.

### Silene fetlerii

D. Pavlova

1D00E329-6390-5D63-BF79-E405E6DD5086

Silene fetlerii D. Pavlova in Ann. Bot. Fennici 51: 387–393 (2014)

#### Materials

- **Type status:** Other material. **Occurrence:** recordedBy: P. Doumas; **Taxon:** scientificName: Silenefetlerii; family: Caryophyllaceae; genus: Silene; specificEpithet: fetlerii; taxonRank: species; **Location:** continent: Europe; country: Greece; stateProvince: Nomos Xanthis; verbatimLocality: ca. 2.4 km SE of Potamochori Village; verbatimElevation: 553 m; verbatimLatitude: 41°14′; verbatimLongitude: 25°04′; **Identification:** identifiedBy: P. Doumas, K. Goula & Th. Constantinidis; **Event:** eventDate: 15 May 2021; habitat: scrub, on serpentine; **Record Level:** collectionID: 18; institutionCode: ATHU; basisOfRecord: Specimen

#### Taxon discussion

*Silenefetlerii* is a new addition to the Greek flora.

This is a rare and local species, recently described from Bulgaria and thought to be confined to the serpentine substrates of only two localities, near the Villages of Fotinovo and Chichevo to the south (Pavlova 2014). Our Greek specimen (Fig. 15) agrees with the description of the Bulgarian material in all essential characters: it has narrow linear leaves and lanceolate bracts, 6-9 mm long calyces, 5-6 mm long capsules included in calyx, a capsule/anthophore ratio of 1.3-2/1 and a rounded capsule base with a narrow and acute neck. We did not observe any viscid indumentum on the lower stem nodes in our specimen, a character usually found in the Bulgarian plants.

This is another species that turns out to cross the political border between Bulgaria and Greece as the new locality lies ca. 12 km south of the border. The new locality expands the known species’ distribution for ca. 25 km to the SW (Fig. 11). This single Greek population fits the Critically Endangered Category [B1ab(i ii iii iv) + 2ab(i ii iii iv)] (IUCN 2012) and shares the same habitat characteristics with that described by Pavlova (2014); even some rare, range-restricted serpentine taxa that accompany *Silenefetlerii* in Bulgaria (Aethionemasaxatilesubsp.rhodopaeum, *Onosmakittanae*, see under these taxa) are also found in Greece. The identity of the *Silenespergulifolia* collections from Nomos Evrou needs further investigation.

### Polygonatum hirtum

(Poir.) Pursh

37CA7196-9C4C-58A1-B88C-FAB17567D029

Polygonatum hirtum (Poir.) Pursh in Fl. Amer. Sept. 1: 234 (1813)

#### Materials

- **Type status:** Other material. **Occurrence:** recordedBy: P. Doumas; **Taxon:** scientificName: Polygonatumhirtum; family: Convallariaceae; genus: Polygonatum; specificEpithet: hirtum; taxonRank: species; **Location:** continent: Europe; country: Greece; stateProvince: Nomos Xanthis; verbatimLocality: Mt. Achladovouno, the north-eastern slopes; verbatimElevation: 606 m; verbatimLatitude: 41°11′; verbatimLongitude: 24°48′; **Identification:** identifiedBy: P. Doumas & K. Goula; **Event:** eventDate: 16 May 2021; habitat: deciduous forest with Carpinus orientalis, limestone; **Record Level:** collectionID: 25; institutionCode: ATHU; basisOfRecord: Specimen

#### Taxon discussion

An uncommon and local species in Greece, recorded from the floristic regions of North Central (NC) and North East (NE; Dimopoulos et al. 2013). This new locality on Mt. Achladovouno is probably the easternmost known so far. Other *Polygonatumhirtum* collections from NE include the Nomos of Chalkidiki (a.n. 1390619 LD), Serres (a.n. 10 0234511, 10 0234512 B) and Kilkis (a.n. 1324835 LD, JACQ-ID 825194, B).

### Convolvulus suendermannii

Bornm.

806119AC-DB65-587F-9183-B5B98EC703A5

Convolvulus suendermannii Bornm. in Repert. Spec. Nov. Regni Veg. 43: 152 (1938)

#### Materials

- **Type status:** Other material. **Occurrence:** recordedBy: P. Doumas; **Taxon:** scientificName: Convolvulussuendermannii; family: Convolvulaceae; genus: Convolvulus ; specificEpithet: suendermannii; taxonRank: species; **Location:** continent: Europe; country: Greece; stateProvince: Nomos Xanthis; verbatimLocality: Mt. Achladovouno, summit area; verbatimElevation: 1395 m; verbatimLatitude: 41°10′; verbatimLongitude: 24°48′; **Identification:** identifiedBy: P. Doumas, K. Goula & Th. Constantinidis; **Event:** eventDate: 4 July 2021; habitat: rocky, calcareous slope; **Record Level:** collectionID: 54; institutionCode: ATHU; basisOfRecord: Specimen

#### Taxon discussion

**Figure 16.**
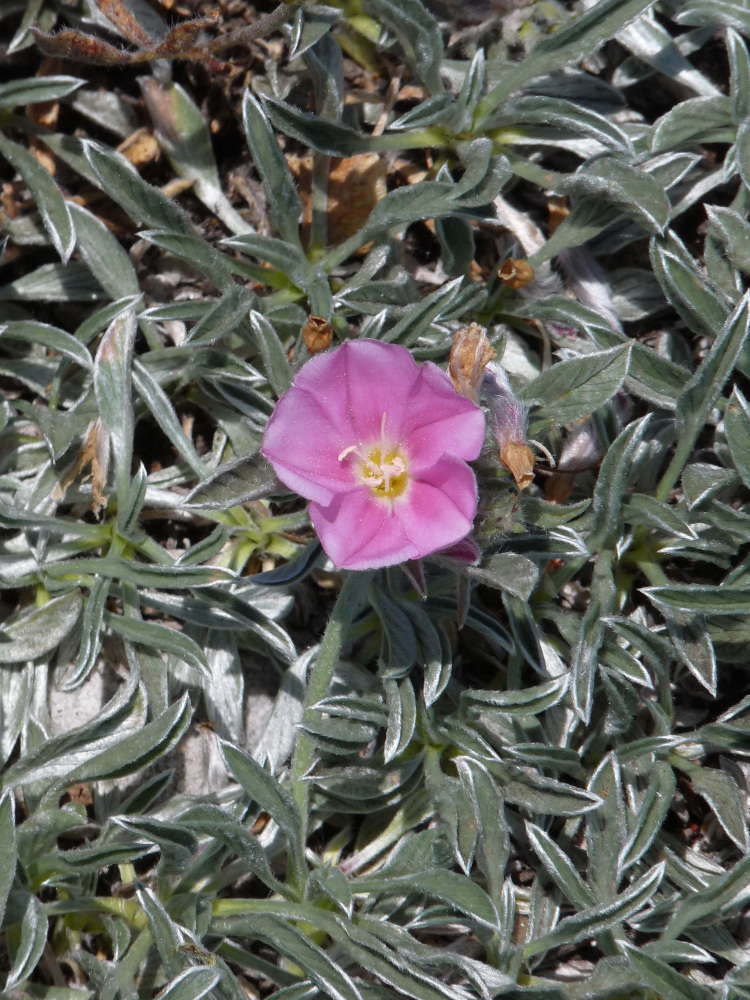
*Convolvulussuendermannii* from Mt. Achladovouno (photo: P. Doumas).

**Figure 17.**
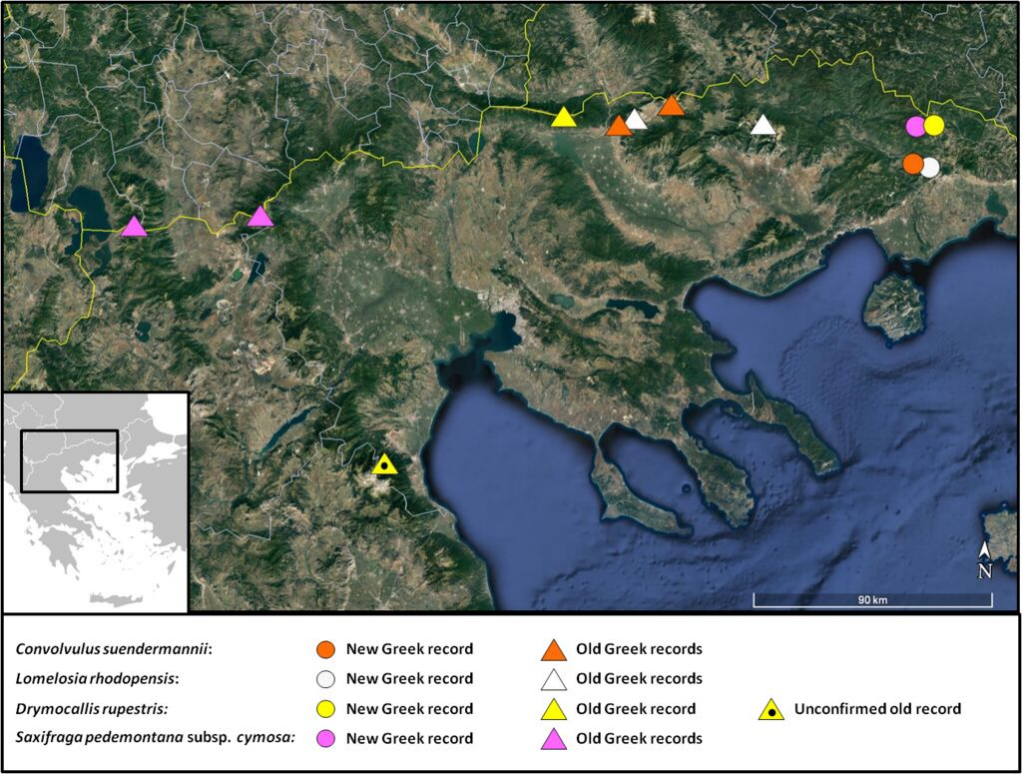
Distribution map of *Convolvulussuendermannii*, *Lomelosiarhodopensis*, *Drymocallisrupestris* and Saxifragapedemontanasubsp.cymosa in Greece.

A very local and range-restricted species, previously known from the Greek and the Bulgarian sides of Mt. Orvilos (also known as Ali Botuš), although Wood et al. (2015) omit Greece in the distribution range of the species. Specimens from Mt. Slavjanka, Bulgaria (Wood et al. 2015) and observations from Mt. Angistro, Greece (Tan and Kofinas 2020) slightly extended the species’ area of occurrence. The new locality on Mt. Achladovouno (Fig. 16), ca. 100 km to the SE of Orvilos, is isolated and forms the easternmost border in the species’ distribution (Fig. 17). It was not found together with either *C.lineatus* L. or C.boissierisubsp.compactus (Boiss.) Stace, the presumed parents of *C.suendermannii* in case the hybridogenous origin is hypothesised.

### Sedum confertiflorum

Boiss.

31011013-4202-5C80-A80D-1185587E3569

Sedum confertiflorum Boiss. in Diagn. Pl. Orient. ser. 1, 3: 15 (1843)

#### Materials

- **Type status:** Other material. **Occurrence:** recordedBy: P. Doumas; **Taxon:** scientificName: Sedumconfertiflorum; family: Crassulaceae; genus: Sedum; specificEpithet: confertiflorum; taxonRank: species; **Location:** continent: Europe; country: Greece; stateProvince: Nomos Evrou; verbatimLocality: 3 km SW of Dadia Village; verbatimElevation: 380 m; verbatimLatitude: 41°06′; verbatimLongitude: 26°11′; **Identification:** identifiedBy: P. Doumas & K. Goula; **Event:** eventDate: 6 May 2013; habitat: rocky area in opening of Pinus forest; **Record Level:** basisOfRecord: Photograph
- **Type status:** Other material. **Occurrence:** recordedBy: K. Goula; **Taxon:** scientificName: Sedumconfertiflorum; family: Crassulaceae; genus: Sedum; specificEpithet: confertiflorum; taxonRank: species; **Location:** continent: Europe; country: Greece; stateProvince: Nomos Evrou; verbatimLocality: 8.5 km SW of Dadia Village; verbatimElevation: 564 m; verbatimLatitude: 41°05′; verbatimLongitude: 26°08′; **Identification:** identifiedBy: K. Goula; **Event:** eventDate: 3 June 2018; habitat: rocky area, on igneous substrate; **Record Level:** collectionID: 2560; institutionCode: ATHU; basisOfRecord: Specimen
- **Type status:** Other material. **Occurrence:** recordedBy: K. Goula; **Taxon:** scientificName: Sedumconfertiflorum; family: Crassulaceae; genus: Sedum; specificEpithet: confertiflorum; taxonRank: species; **Location:** continent: Europe; country: Greece; stateProvince: Nomos Evrou; verbatimLocality: 8.5 km SW of Dadia Village; verbatimElevation: 564 m; verbatimLatitude: 41°05′; verbatimLongitude: 26°08′; **Identification:** identifiedBy: K. Goula; **Event:** eventDate: 20 June 2019; habitat: rocky area, on igneous substrate; **Record Level:** collectionID: 2882; institutionCode: ATHU; basisOfRecord: Specimen

#### Taxon discussion

**Figure 18.**
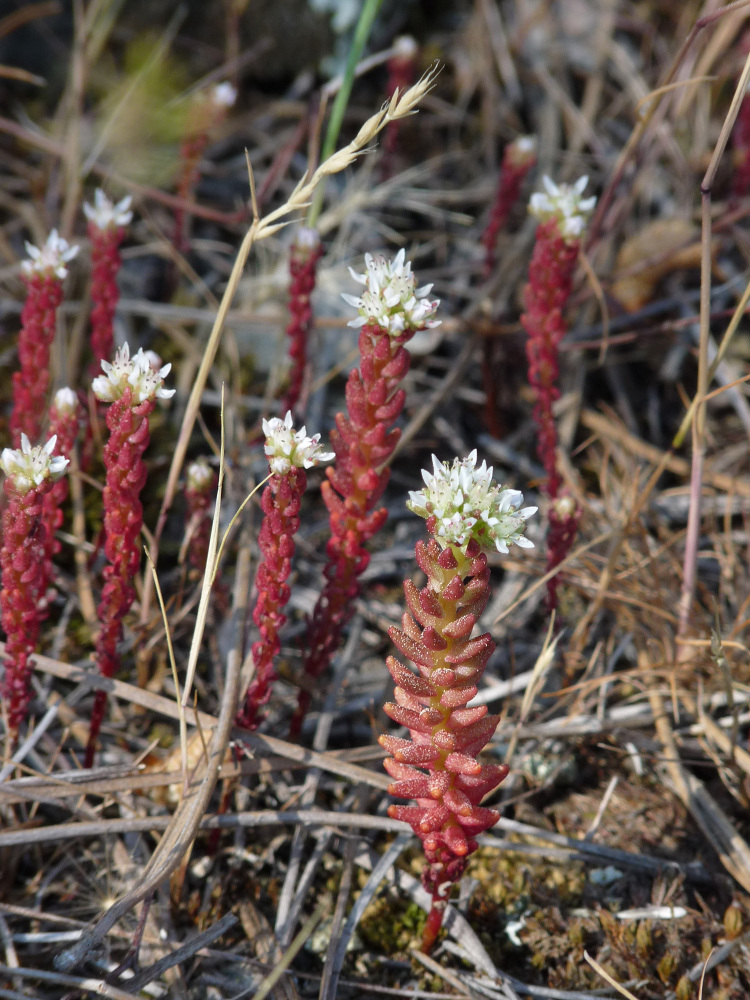
*Sedumconfertiflorum* from Nomos Evrou (photo: P. Doumas).

An east Mediterranean element previously known from Central and West Anatolia (Turkey) and the Greek Island of Lesvos (Chamberlain 1972, Hart 2002). It was recently reported from Bulgaria (Dimitrov 2010), from a locality ca. 60 km NW of the Greek records presented above. The name of the species derives from the characteristic conferted inflorescence. Our collections from Nomos Evrou (Fig. 18) are the first for continental Greece and the floristic region of the NE alike (Fig. 3).

### Lomelosia rhodopensis

(Stoj. & Stef.) Greuter & Burdet

9A226B13-9CE2-5D47-9D3C-727A5EE87D18

Lomelosia rhodopensis (Stoj. & Stef.) Greuter & Burdet in Willdenowia 15(1): 75 (1985)

#### Materials

- **Type status:** Other material. **Occurrence:** recordedBy: P. Doumas; **Taxon:** scientificName: Lomelosiarhodopensis; family: Dipsacaceae; genus: Lomelosia; specificEpithet: rhodopensis; taxonRank: species; **Location:** continent: Europe; country: Greece; stateProvince: Nomos Xanthis; verbatimLocality: Mt. Achladovouno, ca. 300 m NE of the main summit; verbatimElevation: 1314 m; verbatimLatitude: 41°10′; verbatimLongitude: 24°48′; **Identification:** identifiedBy: P. Doumas & K. Goula; **Event:** eventDate: 4 July 2021; habitat: rocky, calcareous slope; **Record Level:** collectionID: 51; institutionCode: ATHU; basisOfRecord: Specimen

#### Taxon discussion

*Lomelosiarhodopensis* is a rare, range-restricted species endemic to NE Greece and south Bulgaria (Kokkini 1991). Greek specimens had been collected only from Mt. Falakro (Kokkini 1991), while an observation from Nomos Serron (Tan and Kofinas 2020) added a second locality. This is the third confirmed population found in the Greek territory (Fig. 17).

### Scabiosa balcanica

Velen.

BF875FA5-360C-5429-ABFF-0B2EACAF413A

Scabiosa balcanica Velen. in Fl. Bulg. 243 (1891)

#### Materials

- **Type status:** Other material. **Occurrence:** recordedBy: P. Doumas; **Taxon:** scientificName: Scabiosabalcanica; family: Dipsacaceae; genus: Scabiosa; specificEpithet: balcanica; taxonRank: species; **Location:** continent: Europe; country: Greece; stateProvince: Nomos Xanthis; verbatimLocality: Mt. Achladovouno, ca. 550 m NE of the main summit; verbatimElevation: 1220 m; verbatimLatitude: 41°10′; verbatimLongitude: 24°48′; **Identification:** identifiedBy: P. Doumas & K. Goula; **Event:** eventDate: 4 July 2021; habitat: rocky, calcareous slope; **Record Level:** collectionID: 52; institutionCode: ATHU; basisOfRecord: Specimen

#### Taxon discussion

*Scabiosabalcanica* is a range-restricted Balkan endemic species distributed in Greece, Bulgaria, North Macedonia and Serbia (Petrova and Vladimirov 2010, Dimopoulos et al. 2013, Tomović et al. 2014). The only report from the Greek floristic region of the NE seems to be on Mt. Falakro (Kokkini 1991). This is the second population discovered in the NE region of Greece.

### Euphorbia carniolica

Jacq.

9DF74888-16FF-500C-B05D-5D3EE7E902A4

Euphorbia carniolica Jacq. in Fl. Austriac. 5(App.): 34, t. 14 (1778)

#### Materials

- **Type status:** Other material. **Occurrence:** recordedBy: P. Doumas; **Taxon:** scientificName: Euphorbiacarniolica; family: Euphorbiaceae; genus: Euphorbia; specificEpithet: carniolica; taxonRank: species; **Location:** continent: Europe; country: Greece; stateProvince: Nomos Xanthis; verbatimLocality: ca. 1.2 km NW of Kotili Village; verbatimElevation: 627 m; verbatimLatitude: 41°20′; verbatimLongitude: 24°52′; **Identification:** identifiedBy: P. Doumas, K. Goula & Th. Constantinidis; **Event:** eventDate: 24 April 2021; habitat: Quercus forest, next to a small stream; **Record Level:** collectionID: 5; institutionCode: ATHU; basisOfRecord: Specimen
- **Type status:** Other material. **Occurrence:** recordedBy: P. Doumas; **Taxon:** scientificName: Euphorbiacarniolica; family: Euphorbiaceae; genus: Euphorbia; specificEpithet: carniolica; taxonRank: species; **Location:** continent: Europe; country: Greece; stateProvince: Nomos Xanthis; verbatimLocality: ca. 1.2 km NW of Kotili Village; verbatimElevation: 627 m; verbatimLatitude: 41°20′; verbatimLongitude: 24°52′; **Identification:** identifiedBy: P. Doumas, K. Goula & Th. Constantinidis; **Event:** eventDate: 11 May 2021; habitat: Quercus forest, next to a small stream; **Record Level:** collectionID: 15; institutionCode: ATHU; basisOfRecord: Specimen

#### Taxon discussion

A new record for the Greek flora (Fig. 19). *Euphorbiacarniolica* is distributed in the area between the Alps (north Italy, Switzerland) and the Eastern Carpathians and extends to Poland and Ukraine to the north (Smith and Tutin 1968). It is considered as a Euro-Siberian element by Geltman (2015) and an Illyricoid element by Trinajstić (1992), but its distribution justifies its placement to the south-east European elements. Τhe populations in the central parts of the Balkans and Romania seem to be the closest to the newly-discovered, quite isolated Greek population. This first Greek record forms the southernmost limit of the species’ distribution.

### Anthyllis aurea

Host

2C4DA2D2-545F-5523-99FC-0F6111E8045E

Anthyllis aurea Host in Fl. Austriac. 2: 319 (1831)

#### Materials

- **Type status:** Other material. **Occurrence:** recordedBy: P. Doumas; **Taxon:** scientificName: Anthyllisaurea; family: Fabaceae; genus: Anthylllis; specificEpithet: aurea; taxonRank: species; **Location:** continent: Europe; country: Greece; stateProvince: Nomos Xanthis; verbatimLocality: Mt. Achladovouno, just below the summit ridge; verbatimElevation: 1343 m; verbatimLatitude: 41°10′; verbatimLongitude: 24°47′; **Identification:** identifiedBy: P. Doumas & K. Goula; **Event:** eventDate: 4 July 2021; habitat: rocky, calcareous slope; **Record Level:** collectionID: 47; institutionCode: ATHU; basisOfRecord: Specimen

#### Taxon discussion

A Balkan Peninsula endemic known to occur in the northern parts of Greece, with Mt. Pangeo, Mt. Orvilos, Mt. Menikio and Mt. Falakro shaping its distribution to the NE (Akeroyd 1986). The new population on Mt. Achladovouno is the easternmost known record in the Greek territory.

### Onobrychis alba calcarea

(Vandas) P.W. Ball 1968

736BCE71-389D-55DB-9E10-7795D39D216B

Onobrychis alba subsp. calcarea (Vandas) P.W. Ball in Feddes Repert. 79: 41 (1968)

#### Materials

- **Type status:** Other material. **Occurrence:** recordedBy: P. Doumas; **Taxon:** scientificName: Onobrychis alba subsp. calcarea; family: Fabaceae; genus: Onobrychis; specificEpithet: alba; infraspecificEpithet: calcarea; taxonRank: subspecies; **Location:** continent: Europe; country: Greece; stateProvince: Nomos Xanthis; verbatimLocality: Mt. Achladovouno, 600 m NE of the main summit; verbatimElevation: 1219 m; verbatimLatitude: 41°10′; verbatimLongitude: 24°48′; **Identification:** identifiedBy: P. Doumas & K. Goula; **Event:** eventDate: 4 July 2021; habitat: rocky, calcareous slope; **Record Level:** collectionID: 49; institutionCode: ATHU; basisOfRecord: Specimen

#### Taxon discussion

A subspecies endemic to the Balkan Peninsula (Strid 1986, Aybeke and Dane 2007). In the floristic region of NE Greece, there are records from Mt. Pangeo, Mt. Menikio, Mt. Falakro, Mt. Orvilos and Mt. Lekani. The new locality on Mt. Achladovouno forms the easternmost border of its distribution range in Greece.

### Gladiolus imbricatus

L.

1EE5DD2F-F2AA-5FFE-AA88-EA07246B9A72

Gladiolus imbricatus L. in Sp. Pl. 1: 37 (1753)

#### Materials

- **Type status:** Other material. **Occurrence:** recordedBy: K. Goula & K. Polymenakos; **Taxon:** scientificName: Gladiolusimbricatus; family: Iridaceae; genus: Gladiolus; specificEpithet: imbricatus; taxonRank: species; **Location:** continent: Europe; country: Greece; stateProvince: Nomos Imathias; verbatimLocality: Mt. Vermio, ca. 5.5 km S of Ano Grammatiko Village; verbatimElevation: 1374 m; verbatimLatitude: 40°40′; verbatimLongitude: 21°56′; **Identification:** identifiedBy: K. Goula; **Event:** eventDate: 14 July 2018; habitat: wet meadow in opening of mixed Fagus sylvatica-Pinus nigra forest; **Record Level:** collectionID: 2704; institutionCode: ATHU; basisOfRecord: Specimen

#### Taxon discussion

This is a species with an European distribution (Hamilton 1980), known locally from the floristic regions of Southern Pindos (SPi), Northern Pindos (NPi), Sterea Ellas (StE) and North East (NE; Dimopoulos et al. 2013). The population on Mt. Vermio is the first recorded from the floristic region of North Central (NC) Greece. Plants from this new population are rather slender, with congested inflorescences consisting of 3-6 flowers.

### Pedicularis friderici-augusti

Tomm.

44C6652E-FEC1-512D-B4F9-89AFDA4FA739

Pedicularis friderici-augusti Tomm. in Linnaea 13(1): 74, t. 2 (1839)

#### Materials

- **Type status:** Other material. **Occurrence:** recordedBy: P. Doumas; **Taxon:** scientificName: Pedicularisfriderici-augusti; family: Orobanchaceae; genus: Pedicularis; specificEpithet: friderici-augusti; taxonRank: species; **Location:** continent: Europe; country: Greece; stateProvince: Nomos Xanthis; verbatimLocality: Mt. Achladovouno, 550 m NE of the main summit; verbatimElevation: 1223 m; verbatimLatitude: 41°10′; verbatimLongitude: 24°48′; **Identification:** identifiedBy: P. Doumas & K. Goula; **Event:** eventDate: 16 May 2021; habitat: rocky, calcareous slope; **Record Level:** collectionID: 21; institutionCode: ATHU; basisOfRecord: Specimen

#### Taxon discussion

Endemic to the Balkan Peninsula and Italy (Bartolucci et al. 2018), *Pedicularisfriderici-augusti* is rare in Greece, as it has been recorded so far from only three mountains of the NE floristic region: Menikio, Falakro and Orvilos (Raus 1991). This is the fourth record in the Greek territory, on a different mountain and the easternmost border of the species’ distribution in the country.

### Pedicularis orthantha

Griseb.

D504540F-7A3C-56AE-8F69-1C6D143A9A21

Pedicularis orthantha Griseb. in Spic. Fl. Rumel. 2(4): 15 (1844)

#### Materials

- **Type status:** Other material. **Occurrence:** recordedBy: P. Doumas; **Taxon:** scientificName: Pedicularisorthantha; family: Orobanchaceae; genus: Pedicularis; specificEpithet: orthantha; taxonRank: species; **Location:** continent: Europe; country: Greece; stateProvince: Nomos Xanthis; verbatimLocality: Mt. Rodopi, area of Koula; verbatimElevation: 1565 m; verbatimLatitude: 41°19′; verbatimLongitude: 24°49′; **Identification:** identifiedBy: P. Doumas & K. Goula; **Event:** eventDate: 23 May 2021; habitat: rocky places, in openings of forest with Pinus peuce; **Record Level:** collectionID: 33; institutionCode: ATHU; basisOfRecord: Specimen

#### Taxon discussion

**Figure 20.**
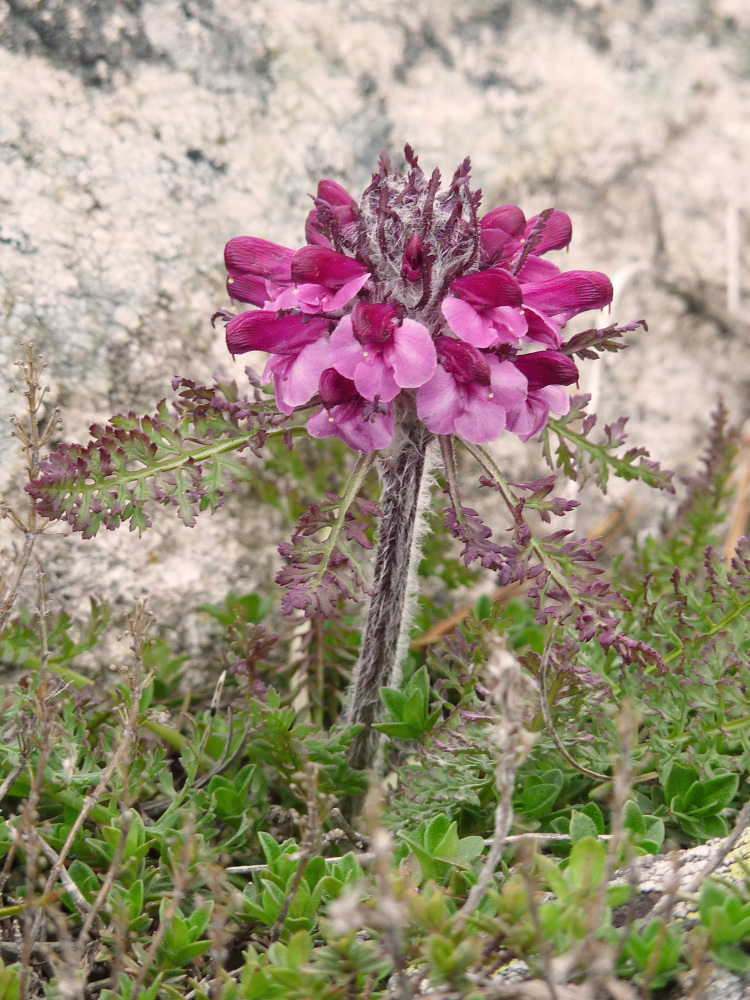
*Pedicularisorthantha* from Mt. Rodopi (photo: P. Doumas).

A range-restricted Balkan endemic with a very limited distribution in Greece, where only two records from the mountains of Voras (NC Greece) and Falakro (NE Greece) are known (Raus 1991). This is the third Greek population (Fig. 20) and the easternmost border of the species distribution in the country.

### Ranunculus platanifolius

L.

30BB0CB2-C612-5355-BA34-2F4B10D5DE77

Ranunculus platanifolius L. in Mantissa 79 (1767)

#### Materials

- **Type status:** Other material. **Occurrence:** recordedBy: K. Goula & C. Dimadis; **Taxon:** scientificName: Ranunculusplatanifolius; family: Ranunculaceae; genus: Ranunculus; specificEpithet: platanifolius; taxonRank: species; **Location:** continent: Europe; country: Greece; stateProvince: Nomos Grevenon; verbatimLocality: Mt. Vasilitsa, ca. 1 km on the way to Samarina after (NW of) the junction that connects Samarina-Smixi and Smixi-Vassilitsa ski centre roads; verbatimElevation: 1642 m; verbatimLatitude: 40°03′; verbatimLongitude: 21°05′; **Identification:** identifiedBy: K. Goula; **Event:** eventDate: 20 June 2020; habitat: damp meadow in Fagus sylvatica forest, serpentine; **Record Level:** collectionID: 3135; institutionCode: ATHU; basisOfRecord: Specimen

#### Taxon discussion

*Ranunculusplatanifolius* is widespread in Europe, but in Greece, it is restricted to a few mountains in the north (Dimopoulos et al. 2013, Strid et al. 2020). In the floristic region of North Pindos, *R.platanifolius* has been recorded from the mountains of Timfi, Smolikas and Gramos (Strid 1986). This locality on Mt. Vasilitsa adds a new mountain massif to the distribution of the species.

### Drymocallis rupestris

(L.) Soják

C2F6DBCA-3A52-5836-8091-2327ED5343E9

Drymocallis rupestris (L.) Soják in Čas. Nár. Muz. Praze, Rada Přír. 154(3-4): 118 (1989)

#### Materials

- **Type status:** Other material. **Occurrence:** recordedBy: P. Doumas; **Taxon:** scientificName: Drymocallisrupestris; family: Rosaceae; genus: Drymocallis; specificEpithet: rupestris; taxonRank: species; **Location:** continent: Europe; country: Greece; stateProvince: Nomos Xanthis; verbatimLocality: Mt. Rodopi, area of Koula; verbatimElevation: 1540 m; verbatimLatitude: 41°19′; verbatimLongitude: 24°49′; **Identification:** identifiedBy: P. Doumas, K. Goula & Th. Constantinidis; **Event:** eventDate: 23 May 2021; habitat: rocky places, in openings of forest with Pinus peuce; **Record Level:** collectionID: 31; institutionCode: ATHU; basisOfRecord: Specimen

#### Taxon discussion

**Figure 21.**
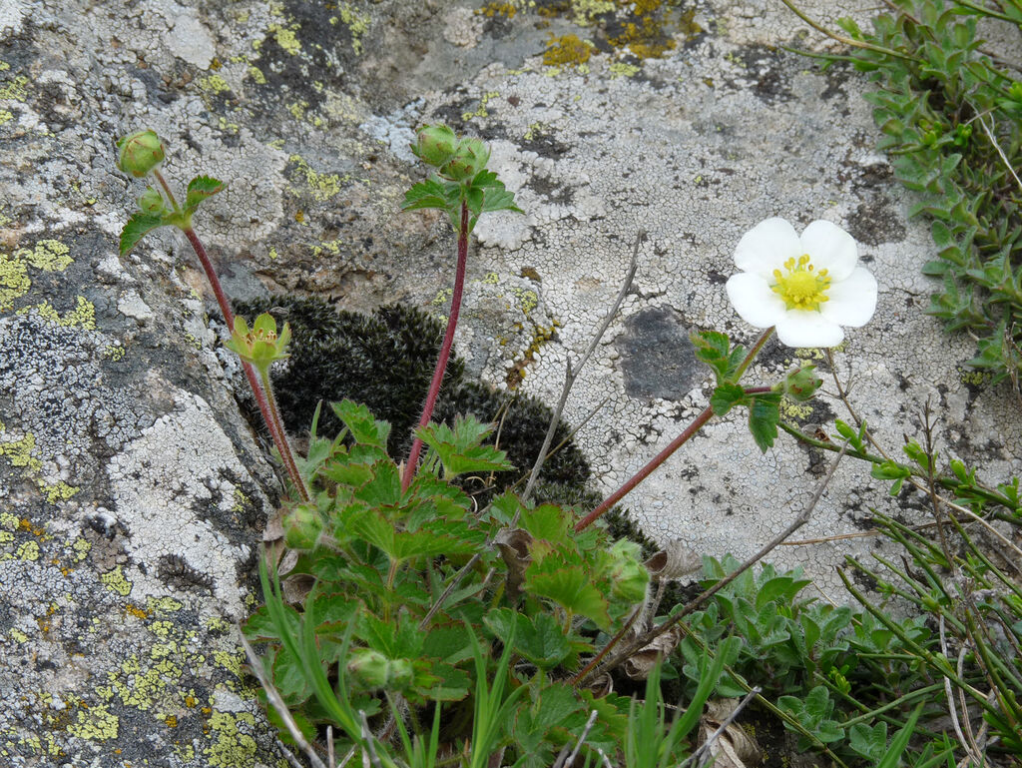
*Drymocallisrupestris* from Mt Rodopi (photo: P. Doumas).

A very rare species in the Greek territory. There is only one previously confirmed record from Mt. Belles (also known as Mt. Kerkini) by Strid (2012), while a historical collection on Mt. Olimbos by Pichler in 1874 may need confirmation (Fig. 17). This new locality from the Rodopi Mountain range comes not as a surprise, as *Drymocallisrupestris* has been recorded from the Bulgarian part of the same mountain (Soják 2011). Our collected plants (Fig. 21) have short stems 10-16 cm high (in contrast to the 15-60 cm high stems of the central European collections) and a rather variable indumentum on leaf petioles and pedicels consisting of mostly patent hairs that become obliquely-patent below the flowers. Morphologically, they may represent transition forms between subsp. rupestris and subsp. banatica (Th. Wolf) Soják. The Rodopi plants grow on rocky slopes, in openings of pine forest and, because of their small size, resemble the Bulgarian dwarf specimens (5-10 cm tall) of subsp. rupestris discussed by Soják (2011). Presumably, the adaptation to rocky substrates and a more xeric environment are responsible for the dwarf habit of our *D.rupestris* samples, but also of the related *D.halacsyana* (Degen) Kurtto & Strid found on Mt. Saos of Samothraki Island, ca. 110 km to the south-east.

### Saxifraga ferdinandi-coburgi

Kellerer & Sünd.

E868AD38-4358-51FF-90DC-7EF6FE4242AD

Saxifraga ferdinandi-coburgi Kellerer & Sünd. in Allg. Bot. Z. Syst. 1901: 116 (1901)

#### Materials

- **Type status:** Other material. **Occurrence:** recordedBy: P. Doumas; **Taxon:** scientificName: Saxifragaferdinandi-coburgi; family: Saxifragaceae; genus: Saxifraga; specificEpithet: ferdinandi-coburgi; taxonRank: species; **Location:** continent: Europe; country: Greece; stateProvince: Nomos Xanthis; verbatimLocality: ca. 2.5 km NNW of the abandoned Village of Imera; verbatimElevation: 1190 m; verbatimLatitude: 41°08′; verbatimLongitude: 24°45′; **Identification:** identifiedBy: P. Doumas & K. Goula; **Event:** eventDate: 30 April 2021; habitat: rocky, calcareous slope facing north; **Record Level:** collectionID: 9; institutionCode: ATHU; basisOfRecord: Specimen
- **Type status:** Other material. **Occurrence:** recordedBy: P. Doumas; **Taxon:** scientificName: Saxifragaferdinandi-coburgi; family: Saxifragaceae; genus: Saxifraga; specificEpithet: ferdinandi-coburgi; taxonRank: species; **Location:** continent: Europe; country: Greece; stateProvince: Nomos Xanthis; verbatimLocality: ca. 2.3 km NW of the village of Chrisa; verbatimElevation: 787 m; verbatimLatitude: 41°08′; verbatimLongitude: 24°50′; **Identification:** identifiedBy: P. Doumas & K. Goula; **Event:** eventDate: 16 April 2020; habitat: rocky, calcareous slope; **Record Level:** basisOfRecord: Photograph
- **Type status:** Other material. **Occurrence:** recordedBy: P. Doumas; **Taxon:** scientificName: Saxifragaferdinandi-coburgi; family: Saxifragaceae; genus: Saxifraga; specificEpithet: ferdinandi-coburgi; taxonRank: species; **Location:** continent: Europe; country: Greece; stateProvince: Nomos Xanthis; verbatimLocality: Mt. Achladovouno, below the summit ridge; verbatimElevation: 1341 m; verbatimLatitude: 41°10′; verbatimLongitude: 24°47′; **Identification:** identifiedBy: P. Doumas & K. Goula; **Event:** eventDate: 16 May 2021; habitat: rocky, calcareous slope; **Record Level:** basisOfRecord: Photograph

#### Taxon discussion

A range-restricted Balkan endemic species that occurs only in SW Bulgaria and NE Greece. Normally a plant of high altitude (over 1500 m) and reported so far from four Greek mountains (Falakro, Pangeo, Orvilos, Menikio; Anagnostopoulos and Strid 2002) and three Bulgarian ones (Slavyanka, Pirin, Central Rodopi; Petrova and Vladimirov 2010). Our three new populations appear to be at the easternmost border of the species’s distribution and grow at the unusually low altitude of 787-1341 m.

### Saxifraga pedemontana cymosa

Engl. 1891

2C7DA0C2-187F-5DDE-B5B4-3803EEE20BDB

Saxifraga pedemontana subsp. cymosa Engl. in Engl. & Prantl, Nat. Pflan. 3: (2a) 55 (1891)

#### Materials

- **Type status:** Other material. **Occurrence:** recordedBy: P. Doumas; **Taxon:** scientificName: Saxifraga pedemontana subsp. cymosa; family: Saxifragaceae; genus: Saxifraga; specificEpithet: pedemontana; infraspecificEpithet: cymosa; taxonRank: subspecies; **Location:** continent: Europe; country: Greece; stateProvince: Nomos Xanthis; verbatimLocality: Mt. Rodopi, area of Koula; verbatimElevation: 1540 m; verbatimLatitude: 41°19′; verbatimLongitude: 24°49′; **Identification:** identifiedBy: P. Doumas & K. Goula; **Event:** eventDate: 23 May 2021; habitat: ledges and fissures on north facing cliffs, in openings of forests with Pinus peuce; **Record Level:** collectionID: 34; institutionCode: ATHU; basisOfRecord: Specimen

#### Taxon discussion

**Figure 22.**
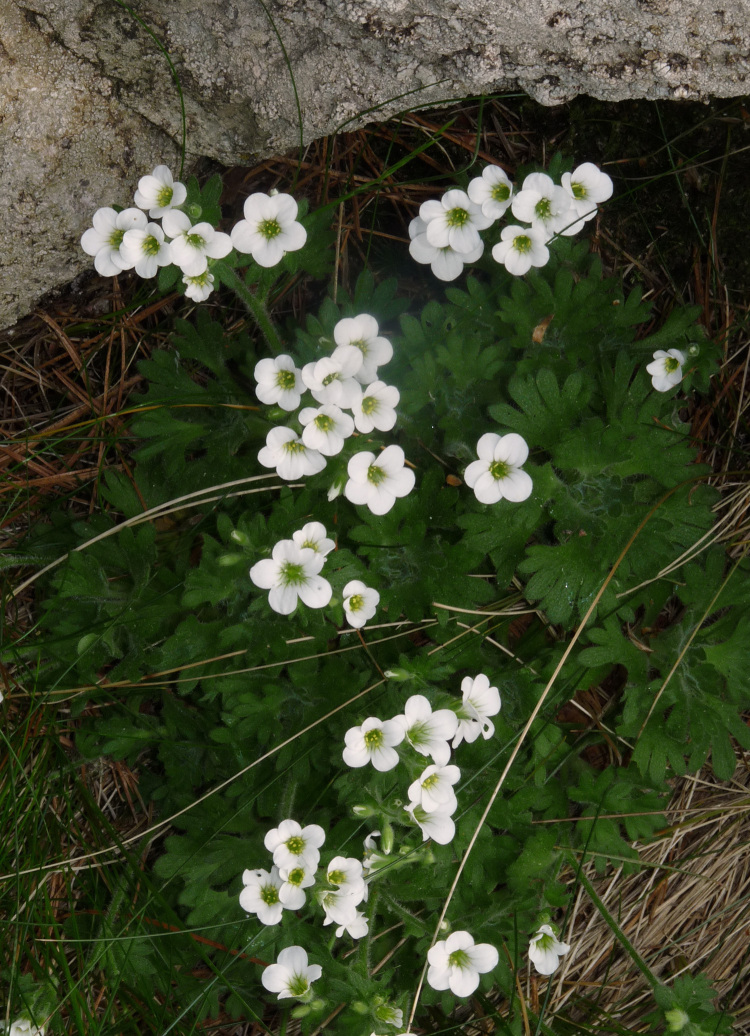
Saxifragapedemontanasubsp.cymosa from Mt. Rodopi (photo: P. Doumas).

In Greece, this subspecies is reported only from the floristic region of NC Greece, where it occurs on Mt. Voras (also known as Kaimaktsalan) and Mt. Varnous (Anagnostopoulos and Strid 2002). This is the third report from Greece (Fig. 22) and the first one from the floristic region of the NE (Fig. 17).

### Saxifraga sibirica

L.

BE0E4091-27DA-5B14-917E-D3D936685BA9

Saxifraga sibirica L. in Syst. Nat., ed. 10. 2: 1027 (1759)

#### Materials

- **Type status:** Other material. **Occurrence:** recordedBy: P. Doumas; **Taxon:** scientificName: Saxifragasibirica; family: Saxifragaceae; genus: Saxifraga; specificEpithet: sibirica; taxonRank: species; **Location:** continent: Europe; country: Greece; stateProvince: Nomos Rodopis; verbatimLocality: near abandoned settlement of Astrea; verbatimElevation: 370 m; verbatimLatitude: 41°13′; verbatimLongitude: 25°08′; **Identification:** identifiedBy: P. Doumas & K. Goula; **Event:** eventDate: 11 April 2021; habitat: damp, shady places on sandstone rocks; **Record Level:** collectionID: 1; institutionCode: ATHU; basisOfRecord: Specimen
- **Type status:** Other material. **Occurrence:** recordedBy: P. Doumas; **Taxon:** scientificName: Saxifragasibirica; family: Saxifragaceae; genus: Saxifraga; specificEpithet: sibirica; taxonRank: species; **Location:** continent: Europe; country: Greece; stateProvince: Nomos Evrou; verbatimLocality: ca. 3 km SE of Megalo Derio Village; verbatimElevation: 193 m; verbatimLatitude: 41°13′; verbatimLongitude: 26°03′; **Identification:** identifiedBy: P. Doumas & K. Goula; **Event:** eventDate: 8 May 2021; habitat: rocky area next to a stream; **Record Level:** collectionID: 14; institutionCode: ATHU; basisOfRecord: Specimen

#### Taxon discussion

**Figure 23.**
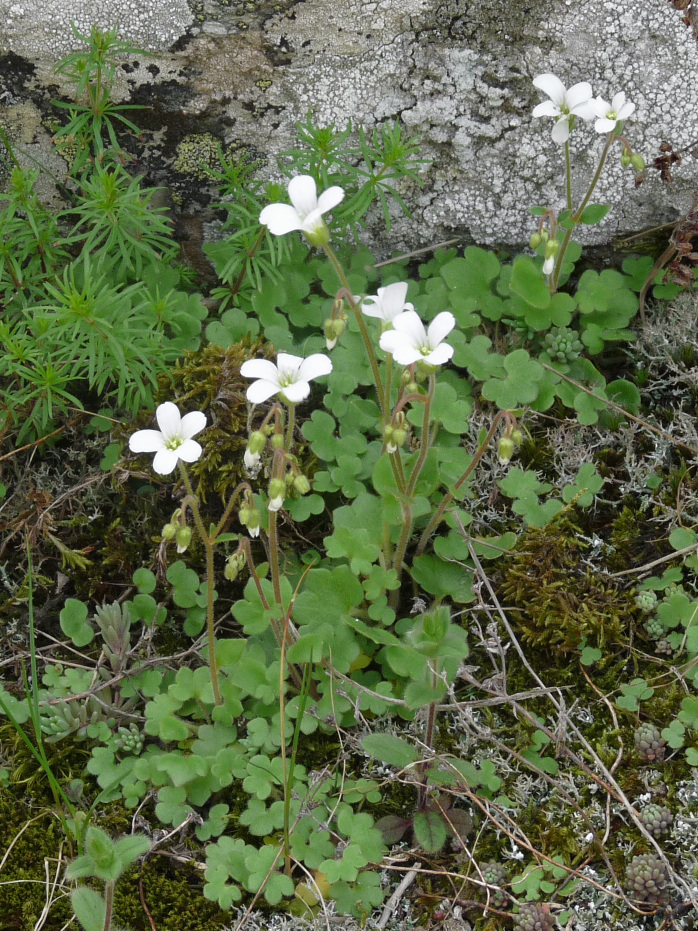
*Saxifragasibirica* from Nomos Evrou (photo: P. Doumas).

*Saxifragasibirica* is rare in Greece, known only from its insular Aegean part (the Islands of Samothraki, Lesvos, Ikaria and Samos; Strid 2016b). The above-cited collections from two adjacent prefectures of Thraki (Fig. 23) are new records for the NE floristic region and continental part of Greece as a whole (Fig. 3).

## Discussion

In this report, we provide new localities for 32 taxa (species or subspecies, see Suppl. material 1) mostly collected in the Greek floristic region of the North East (NE). Two records come from the regions of North Central (NC, *Gladiolusimbricatus*) and North Pindos (NPi, *Ranunculusplatanifolius*), respectively. Seven taxa are new additions to the native Greek flora (Aethionemasaxatilesubsp.rhodopaeum, *Anthericumramosum*, *Asteramellus*, *Euphorbiacarniolica*, Hesperismatronalissubsp.matronalis, *Saponariastrajensis* and *Silenefetlerii*), which now reaches at least 6,712 vascular plants. Three of our records are new additions to the NE floristic region and the Greek mainland alike (*Saxifragasibirica*, *Sedumconfertiflorum* and *Trachomitumvenetum*). A taxonomic correction for Greek *Trachomitum* is proposed and a new identity is attributed to its two known populations. All our records are useful to the “Flora of Greece” project that attempts to enlist, document and present the Greek vascular plant diversity and is currently in progress.

Half of the taxa included in this report have a very limited distribution in Greece, as they had been reported earlier from one to three localities only. Our new localities extend their distribution either within the Greek territory (e.g. *Trachomitumvenetum*, Jacobaeaothonnaesubsp.anomala) or expand their total known range (e.g. *Convolvulussuendermannii*, *Onosmakittanae*). As such, they are important to the ongoing Red Data List project undertaken by the Hellenic Botanical Society that endeavours to attribute each Greek plant taxonomic entity to an IUCN Category (IUCN 2012). Extent of occurrence (EO) and area of occupancy (AOO) for these rare taxa are certainly affected by new localities, particularly marginal ones and several of our records form new distribution borders to the east, west or south. On the same basis, our new positions should be considered in the case of developmental projects or other actions that threaten natural population and degrade or modify the natural habitat of rare plants.

Three additional points of interest can be deduced from our report:

a. The areas adjacent to the borders can be promising for new floristic discoveries in Greece and in different countries as well. All the new country records in this work come from a 1.5 to 12 km zone off the border-line. Often, this same zone has a low inhabitant density and the environment retains much of its naturalness. Road network or vehicle accesses may be limited and, therefore, botanical exploration may have been delayed.

b. When range-restricted plant taxa with a limited area of distribution happen to cross political borders (as, for example, *Lomelosiarhodopensis*, *Saponariastranjensis* and *Saxifragaferdinandi-coburgi* in our case), they are no longer considered single country endemics. For plants, political borders certainly have no geographic sense (Stefanaki et al. 2010). However, these taxa may not be found in national lists of endemic species despite their highly-restricted nature and, therefore, may not have received much conservation attention. For local taxa, considering a distribution range scale of, for example, 10, 50, 100 or 500 km rather than an on/off endemic/non-endemic status can easily fix this discrepancy.

c. Compared to high mountains, mountains of average altitude have received less floristic attention in Greece. Mt. Achladovouno (1,402 m at its highest peak) is such a case with 14 of our records coming from its area and forming distribution limits. This Mountain consists almost exclusively of marble (Bornovas and Rondogianni-Tsiambaou 1983) and is located only 9 km north-west of the City of Xanthi. Until now, it was floristically unexplored. Similar mountains, particularly when close to the border, may be a source of new floristic records, even at country level.

## Supplementary Material

XML Treatment for Anthericum ramosum

XML Treatment for Trachomitum venetum

XML Treatment for Asphodeline taurica

XML Treatment for Aster amellus

XML Treatment for Centaurea marmorea

XML Treatment for Jacobaea othonnae anomala

XML Treatment for Takhtajaniantha austriaca

XML Treatment for Onosma kittanae

XML Treatment for Aethionema saxatile rhodopaeum

XML Treatment for Hesperis matronalis matronalis

XML Treatment for Campanula orphanidea

XML Treatment for Cerastium decalvans orbelicum

XML Treatment for Dianthus petraeus orbelicus

XML Treatment for Petrorhagia cretica

XML Treatment for Saponaria stranjensis

XML Treatment for Silene fetlerii

XML Treatment for Polygonatum hirtum

XML Treatment for Convolvulus suendermannii

XML Treatment for Sedum confertiflorum

XML Treatment for Lomelosia rhodopensis

XML Treatment for Scabiosa balcanica

XML Treatment for Euphorbia carniolica

XML Treatment for Anthyllis aurea

XML Treatment for Onobrychis alba calcarea

XML Treatment for Gladiolus imbricatus

XML Treatment for Pedicularis friderici-augusti

XML Treatment for Pedicularis orthantha

XML Treatment for Ranunculus platanifolius

XML Treatment for Drymocallis rupestris

XML Treatment for Saxifraga ferdinandi-coburgi

XML Treatment for Saxifraga pedemontana cymosa

XML Treatment for Saxifraga sibirica

3EBE1B90-0B0A-5CCD-AA85-FD09947E9949 10.3897/BDJ.10.e81817.suppl1 Supplementary material 1 Examined Material Table Data type occurrences File: oo_639756.xls https://binary.pensoft.net/file/639756 P. Doumas, K. Goula & Th. Constantinidis

## Figures and Tables

**Figure 6a.**
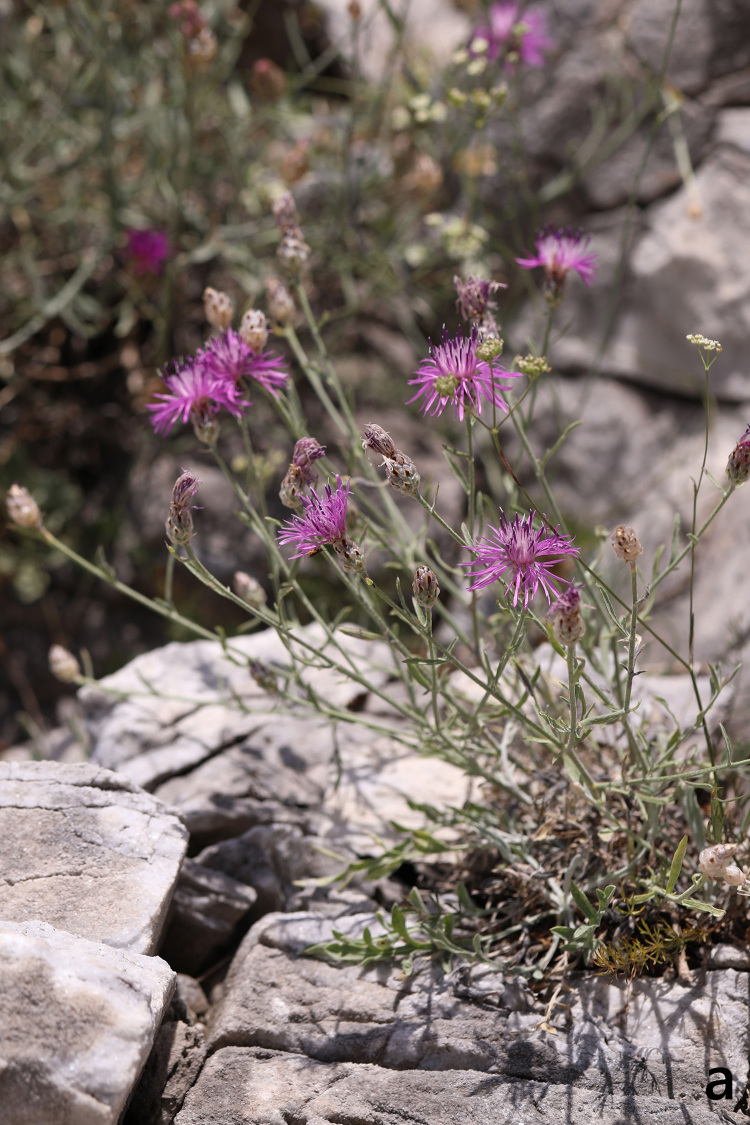
*Centaureamarmorea*, habit (photo: K. Goula).

**Figure 6b.**
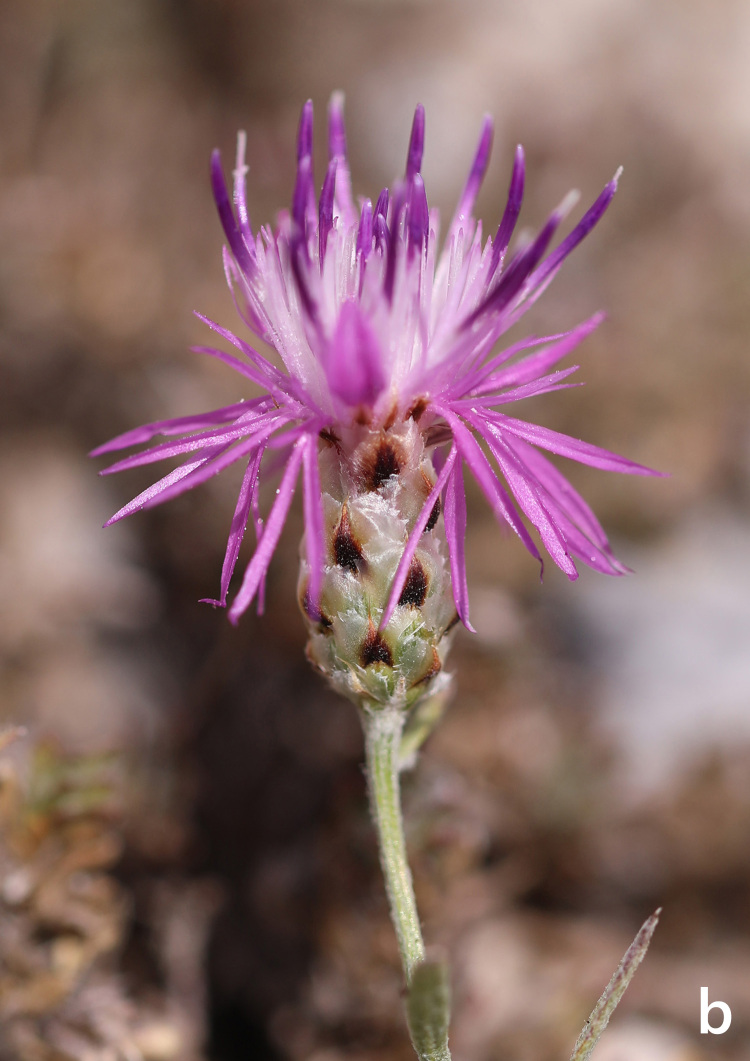
*Centaureamarmorea*, capitulum (photo: K. Goula).

**Figure 12a.**
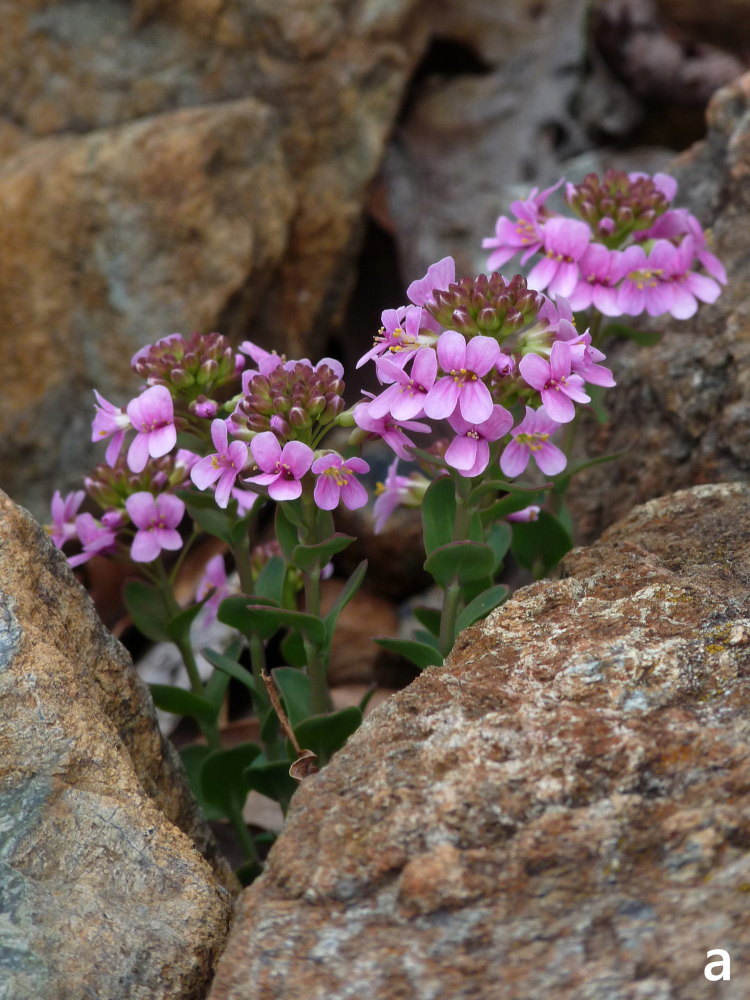
Aethionemasaxatilesubsp.rhodopaeum in flower (photo: P. Doumas).

**Figure 12b.**
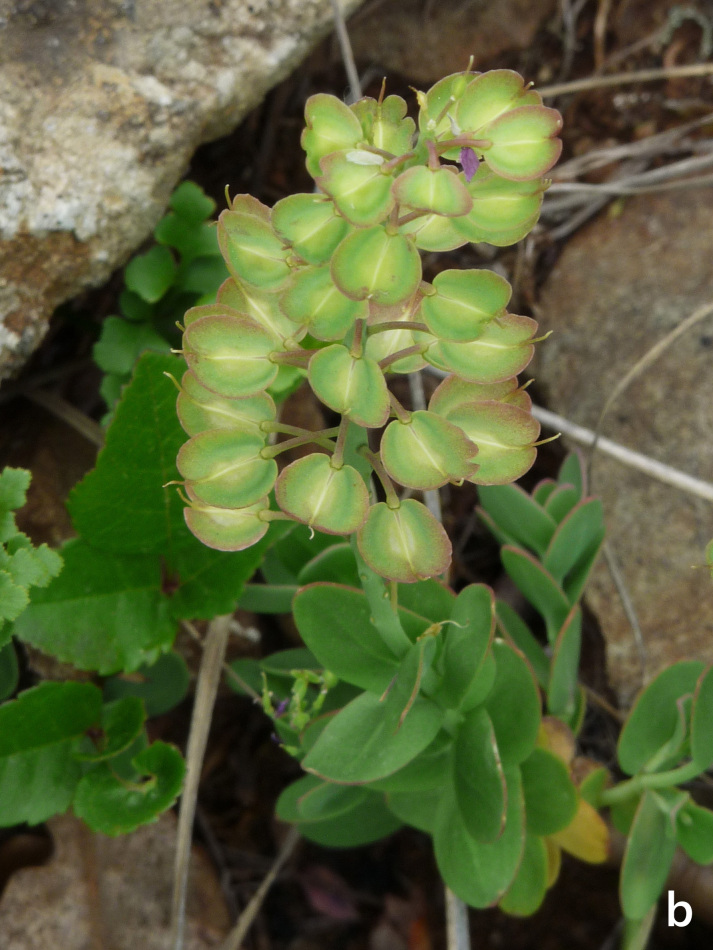
Aethionemasaxatilesubsp.rhodopaeum iin fruit (photo: P. Doumas).

**Figure 13a.**
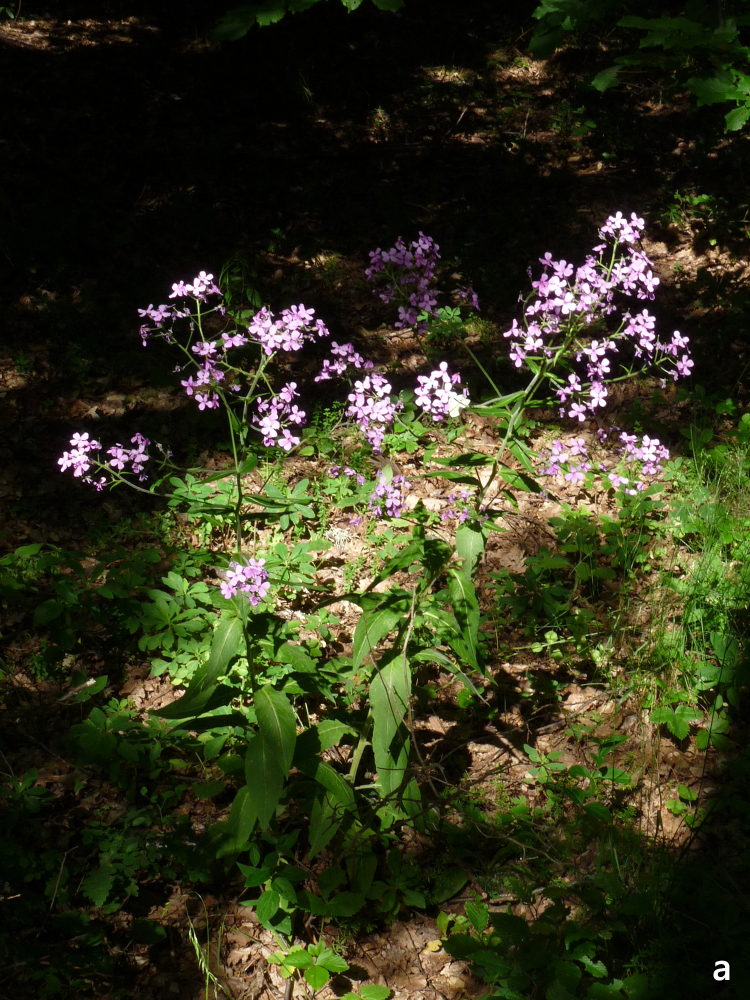
Hesperismatronalissubsp.matronalis, habit (photo: P. Doumas).

**Figure 13b.**
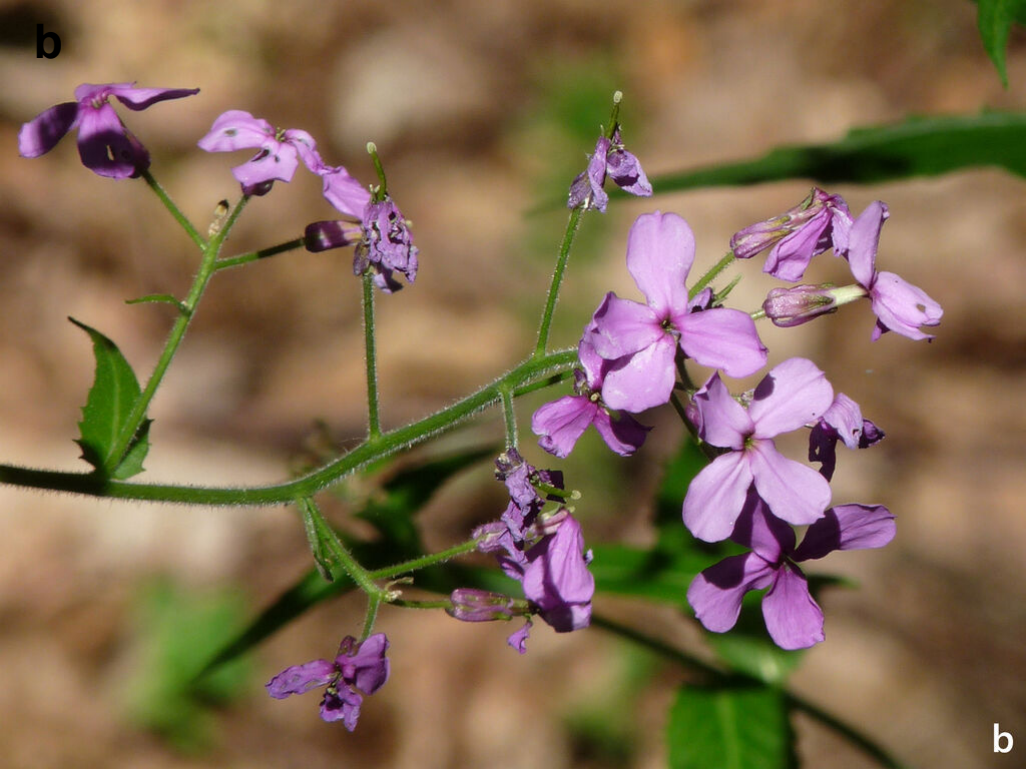
Hesperismatronalissubsp.matronalis, inflorescence (photo: P. Doumas).

**Figure 15a.**
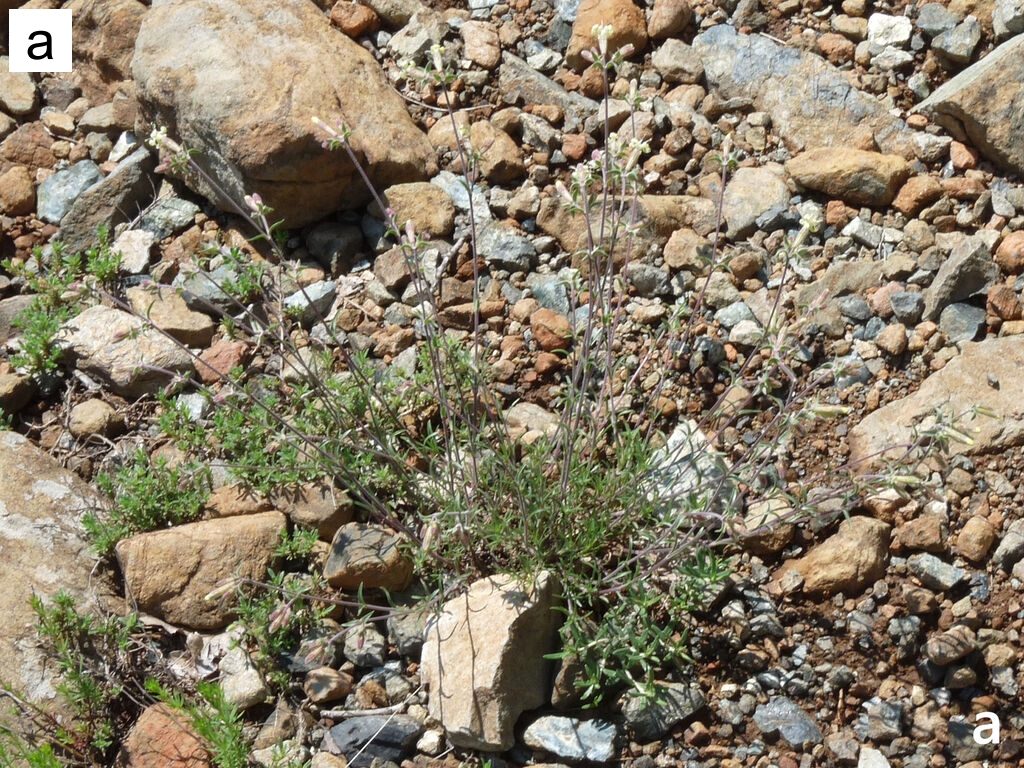
*Silenefetlerii*, habit (photo: P. Doumas).

**Figure 15b.**
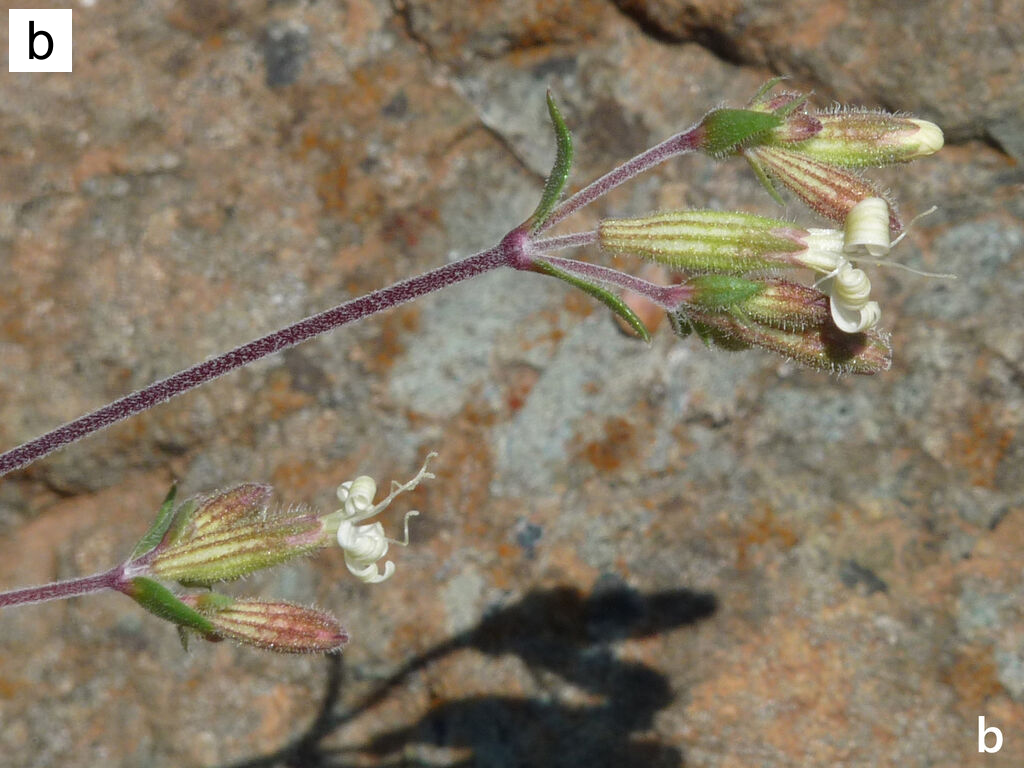
*Silenefetlerii* , inflorescence (photo: P. Doumas).

**Figure 19a.**
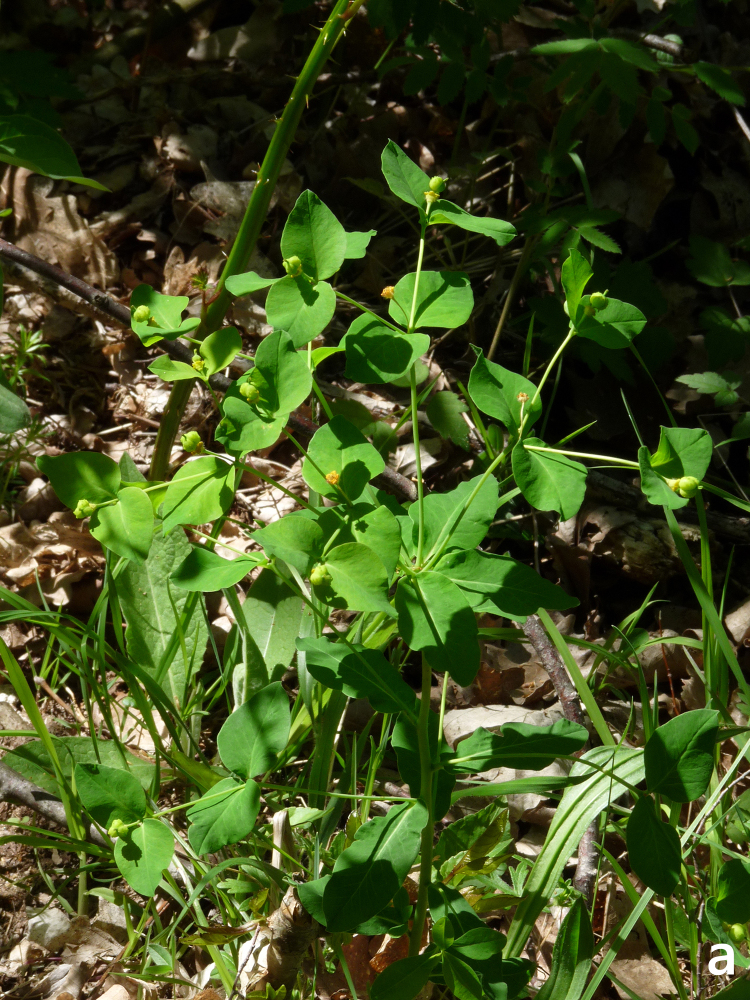
*Euphorbiacarniolica* habit (photo: P. Doumas).

**Figure 19b.**
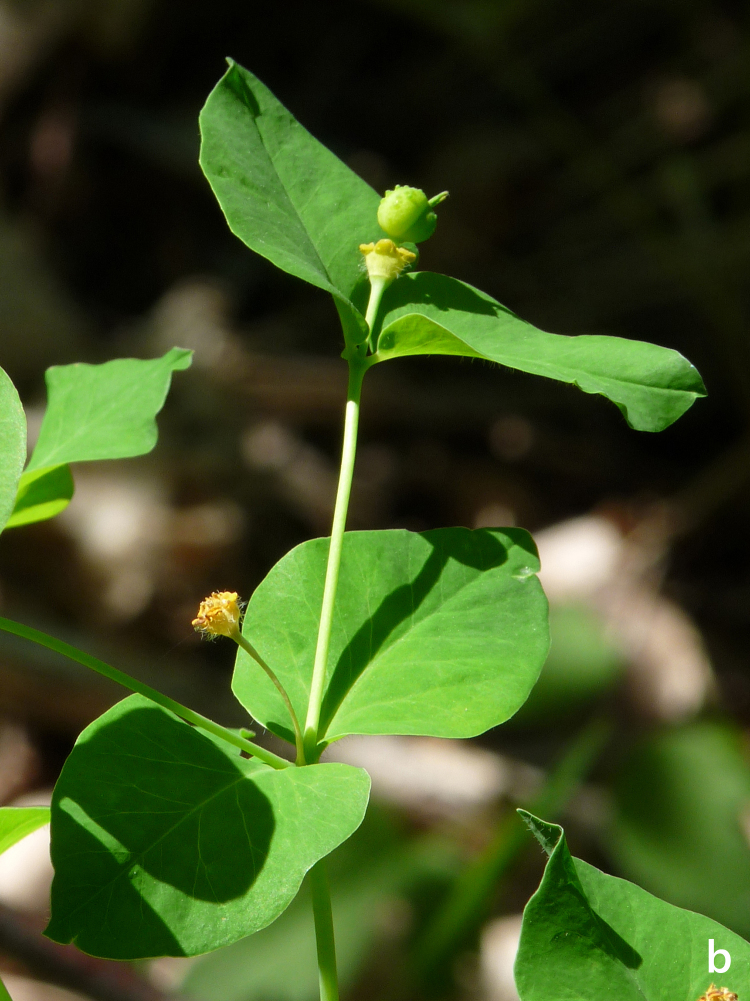
*Euphorbiacarniolica*, inflorescence (photo: P. Doumas).

**Table 1.** Key to the subspecies of *Aethionemasaxatile* found in Greece:

1. A large proportion of fruits (> 30-40%) unilocular. Plants compact, occasionally with a thick woody base up to 1 cm wide; flowering stems usually short, 3-10(-12) cm long	2
1a. Unilocular fruits uncommon (< 30-40%) or absent. At least some stems usually longer; plant less compact, woody base thinner	3
2. Style (1.7-)2-3(-3.3) mm long, stamens without appendages or appendages rudimentary, petals dark pink-purplish	subsp. rhodopaeum
2a. Style 0.3-1.1 mm long, stamens with appendages, petals white to pink	subsp. creticum
3. Unilocular fruits absent; leaves suborbicular to ovate-elliptic, fleshy, less than 3 times as long as wide, up to 23 x 14 mm; petals (4.8-)4.9-7.8(-8) mm long; style length up to 2.1 mm (mean value 1.5 mm)	subsp. corinthiacum
3a. Unilocular fruits often present; leaves elliptic to oblong, not or slightly fleshy, up to 16 x 8 mm and up to 6 times as long as wide; petals (2.8-)3-6.6(-7.7) mm long; style length shorter than 1.8 mm (mean value 0.5-1.0 mm)	4
4. Petals 4-6.6(-7.7) mm long; sepals up to 3.0 mm long; style 0.3-1.6(-1.8) mm long, usually 1.0 mm or longer (mean value 1 mm)	subsp. graecum
4a. Petals 2.8-4.2(-4.7) mm long; sepals shorter than 2.6 mm long; style (0.2-) 0.3-0.9(-1.1) mm long, very rarely longer than 0.9 mm (mean value 0.5 mm)	subsp. oreophilum
